# Supplementary material for: Quantifying epidemiological drivers of gambiense human African Trypanosomiasis across the Democratic Republic of Congo
Source: PLoS Comput Biol. 2021 Jan 29;17(1):e1008532. doi: 10.1371/journal.pcbi.1008532 (PMC7899378; doi:10.1371/journal.pcbi.1008532)
Supplement: S2 Text — Additional results figures and tables. Representations of posterior distributions of fitted parameters for the two example health zones, across the country and within the former provinces of Bandundu and Bas Congo. (PDF) [file pcbi.1008532.s002.pdf]

Supporting Information: Quantifying epidemiological drivers of *gambiense* human African trypanosomiasis across the Democratic Republic of Congo. S2 Posteriors of fitted parameters

Ronald E Crump[1,2,3◑*], Ching-I Huang[1,2◑], Edward S Knock[1,4], Simon E F Spencer[1,4], Paul E Brown[1,2], Erick Mwamba Miaka[5], Chansy Shampa[5], Matt J Keeling[1,2,3], and Kat S Rock[1,2]

[1]Zeeman Institute for System Biology and Infectious Disease Epidemiology Research, The University of Warwick, Coventry, U.K.
[2]Mathematics Institute, The University of Warwick, Coventry, U.K.
[3]The School of Life Sciences, The University of Warwick, Coventry, U.K.
[4]The Department of Statistics, The University of Warwick, Coventry, U.K.
[5]PNLTHA, Kinshasa, D.R.C.

January 19, 2021

◑These authors contributed equally to this work.
* Corresponding author: r.e.crump@warwick.ac.uk

S2.1 Posterior characteristics for example health zones

Table S2.1 shows summaries of posterior distributions of all fitted parameters in two example health zones: Kwamouth in the former Bandundu province and Tandala in the former Equateur province. Kwamouth is categorised as a high-risk health zone and Tandala is at low-risk. Our fitting results show that Kwamouth has higher R_0 and r but lower active screening specificity, reporting rate and passive detection rate than Tandala. These factors provide some possible explanation for why gHAT is more persistent in Kwamouth, as well as having more cases reported every year.

Table S2.1: **Posteriors of fitted parameters.** Notation, brief description, and [2.5th, 50th & 97.5th] percentile of posteriors for fitted parameters.

Notation	Description	Kwamouth	Tandala
R_0	Basic reproduction number (NGM approach)	[1.06, 1.09, 1.15]	[1.006, 1.009, 1.014]
r	Relative bites taken on high-risk humans	[3.06, 6.49, 10.98]	[1.30, 2.04, 4.26]
k_1	Proportion of low-risk people	[0.82, 0.90, 0.95]	[0.85, 0.95, 0.99]
η_H^{post}	Treatment rate from stage 1, 1998 onwards (days^{-1})	[0.60, 1.29, 3.06] $\times 10^{-4}$	[1.11, 2.74, 4.99]$\times 10^{-4}$
γ_H^{post}	Exit rate from stage 2 (treatment or death), 1998 onwards (days^{-1})	[0.42, 1.79, 4.90] $\times 10^{-3}$	[1.72, 3.60, 8.98]$\times 10^{-3}$
$b_{\gamma_H^{\text{pre}}}$	Relative exit rate from stage 2 factor, pre-1998	[0.73, 0.92, 1.00]	[0.54, 0.69, 0.94]
γ_H^{pre}	Exit rate from stage 2 (treatment or death), pre-1998 (days^{-1})	[0.33, 1.64, 4.65] $\times 10^{-3}$	[1.03, 2.53, 7.14]$\times 10^{-3}$
Spec	Active screening diagnostic specificity	[0.9986, 0.9991, 0.9997]	[0.9997, 0.9998, 0.9999]
u	Proportion of stage 2 passive cases reported	[0.18, 0.28, 0.41]	[0.29, 0.39, 0.51]
d_{change}	Midpoint year for passive improvement	[2004.3, 2005.8, 2007.3]	–
$\eta_{H_{\text{amp}}}$	Relative improvement in passive stage 1 detection rate	[0.85, 2.50, 5.74]	–
$\gamma_{H_{\text{amp}}}$	Relative improvement in passive stage 2 detection rate	[0.24, 0.53, 0.99]	–
d_{steep}	Speed of improvement in passive detection rate (years^{-1})	[0.68, 0.94, 1.27]	–

S2.2 Joint posteriors for example health zones

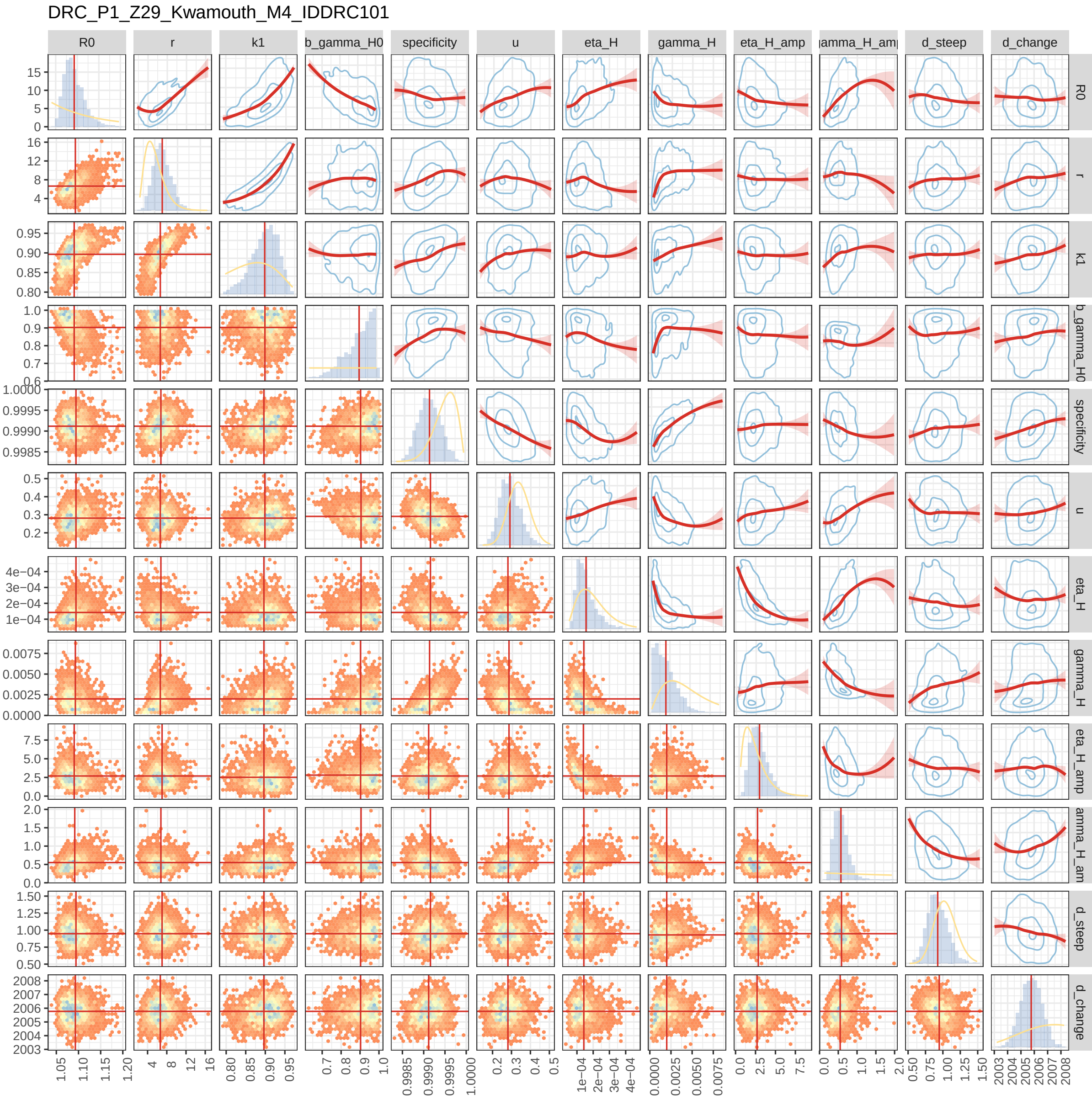

Figure S2.1: Joint posterior distributions for Kwamouth health zone in the former Bandundu province.

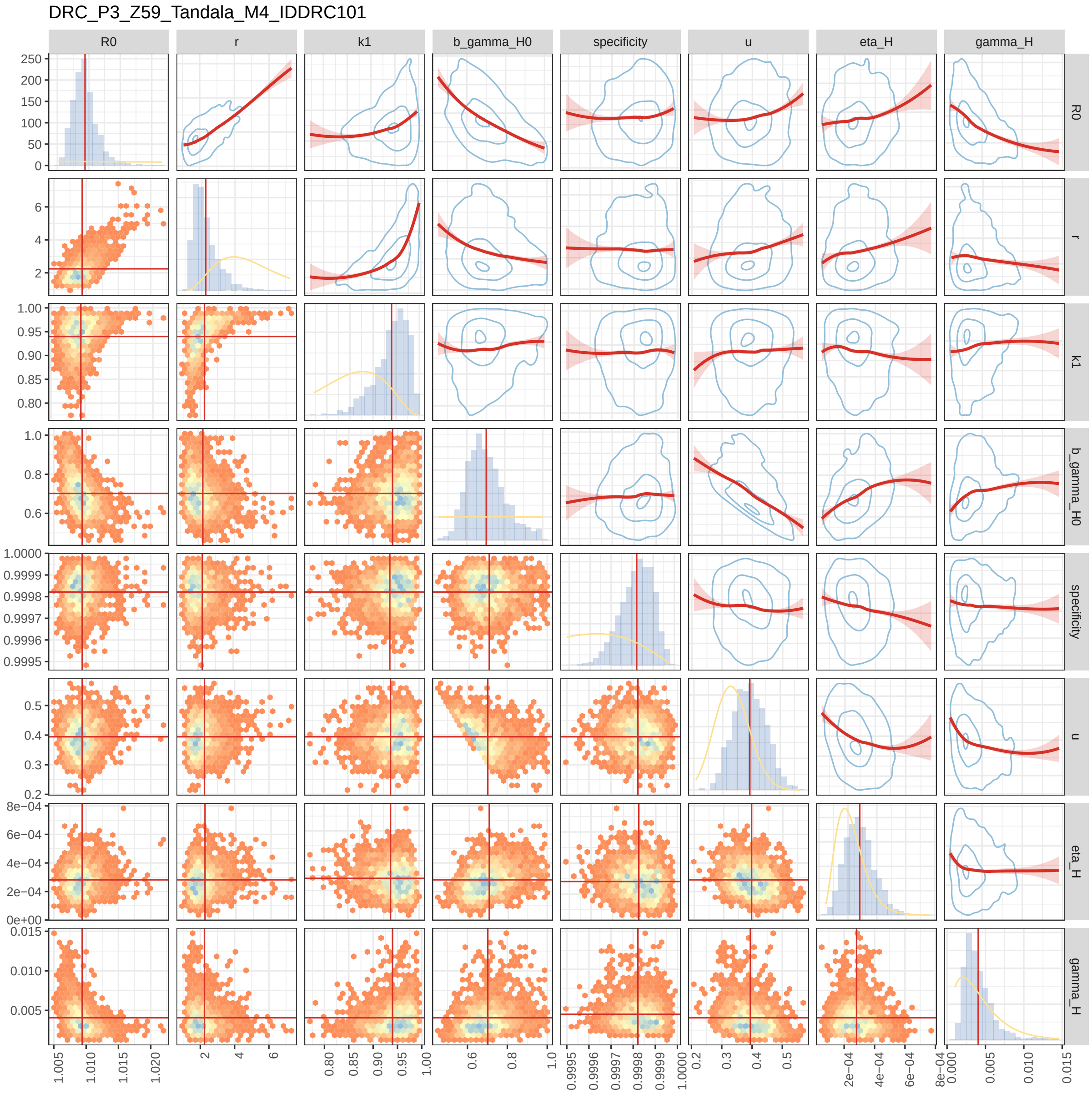

Figure S2.2: Joint posterior distributions for Tandala health zone in the former Equateur province.

S2.3 Posterior distribution maps

The posterior distribution maps (Figure S2.3–S2.16) illustrate both the level and variability of the parameter estimates between health zones. We partition the national or province level map into tessellated equilateral hexagons, or partial hexagons at national and health zone borders, and then fill each of these shapes with colour based on the value of randomly sampled values from the posterior distribution of the parameter being plotted from the analysis of the health zone in which that hexagon, or partial hexagon, lies. In this way, the 'overall' impression of the colour for a health zone gives an indication of the most probable value of the parameter, while the variation in colour reflects the uncertainty in the parameter estimates.

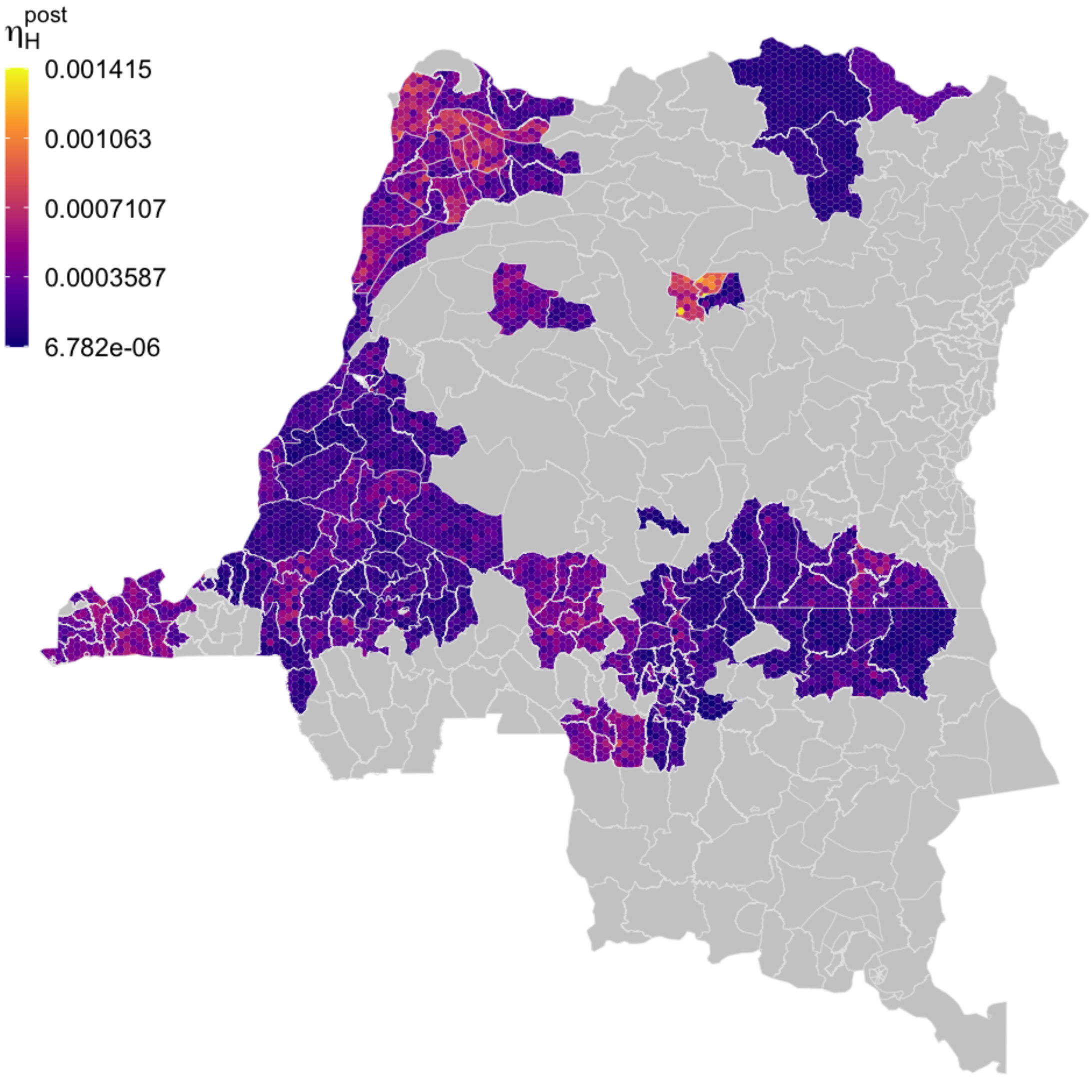

Figure S2.3: Within health zone posterior distribution of η_H^{post}, treatment rate from stage 1, 1998 onwards. fill colours are a randomly sampled from the posterior distribution for the health zone. Shapefiles used to produce these maps are available under an ODC-ODbL licence at `https://data.humdata.org/dataset/drc-health-data`.

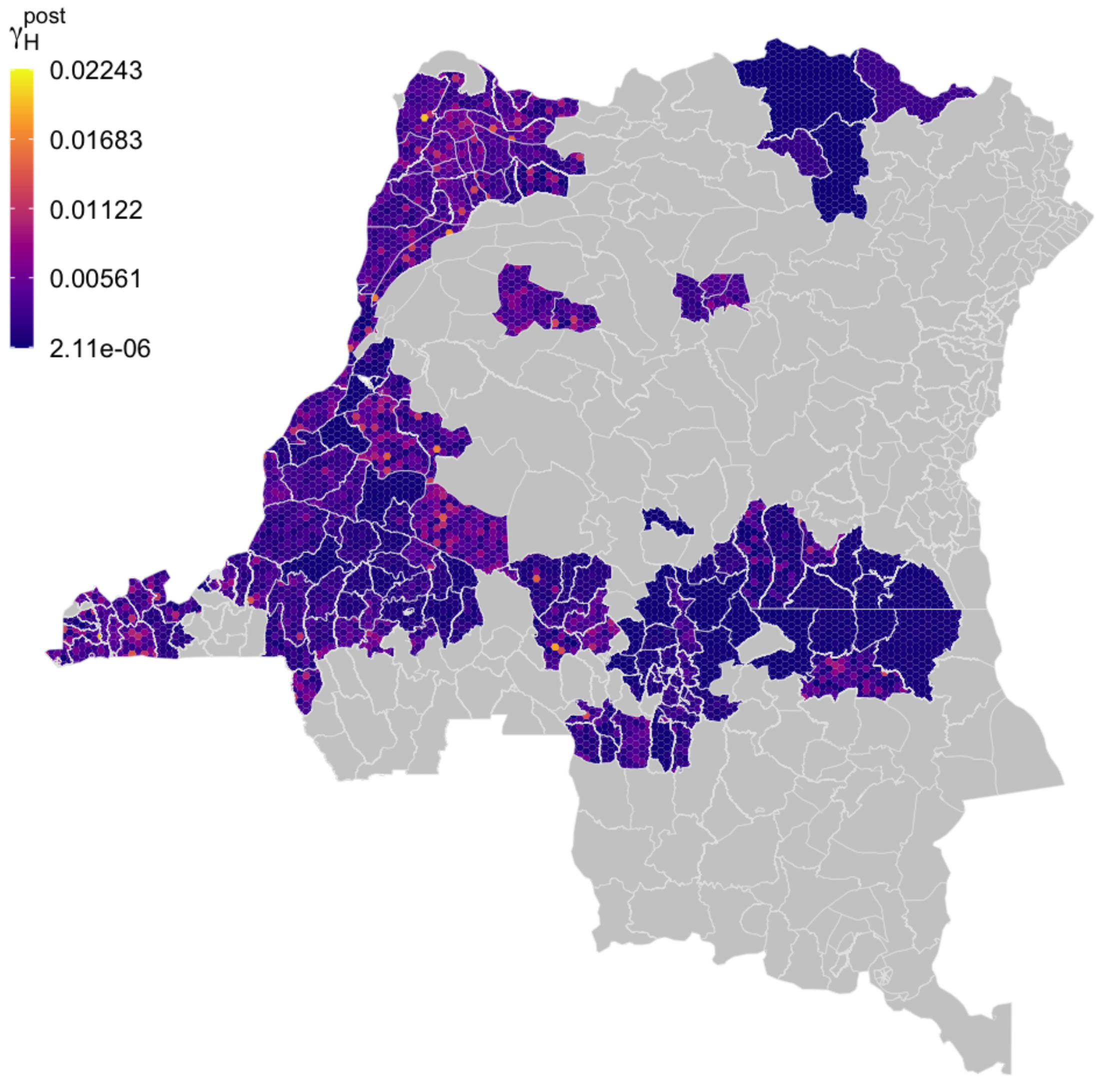

Figure S2.4: Within health zone posterior distribution of γ_H^{post}, the exit rate from stage 2 (treatment or death), 1998 onwards. The fill colours are a randomly sampled from the posterior distribution for the health zone. Shapefiles used to produce these maps are available under an ODC-ODbL licence at `https://data.humdata.org/dataset/drc-health-data`.

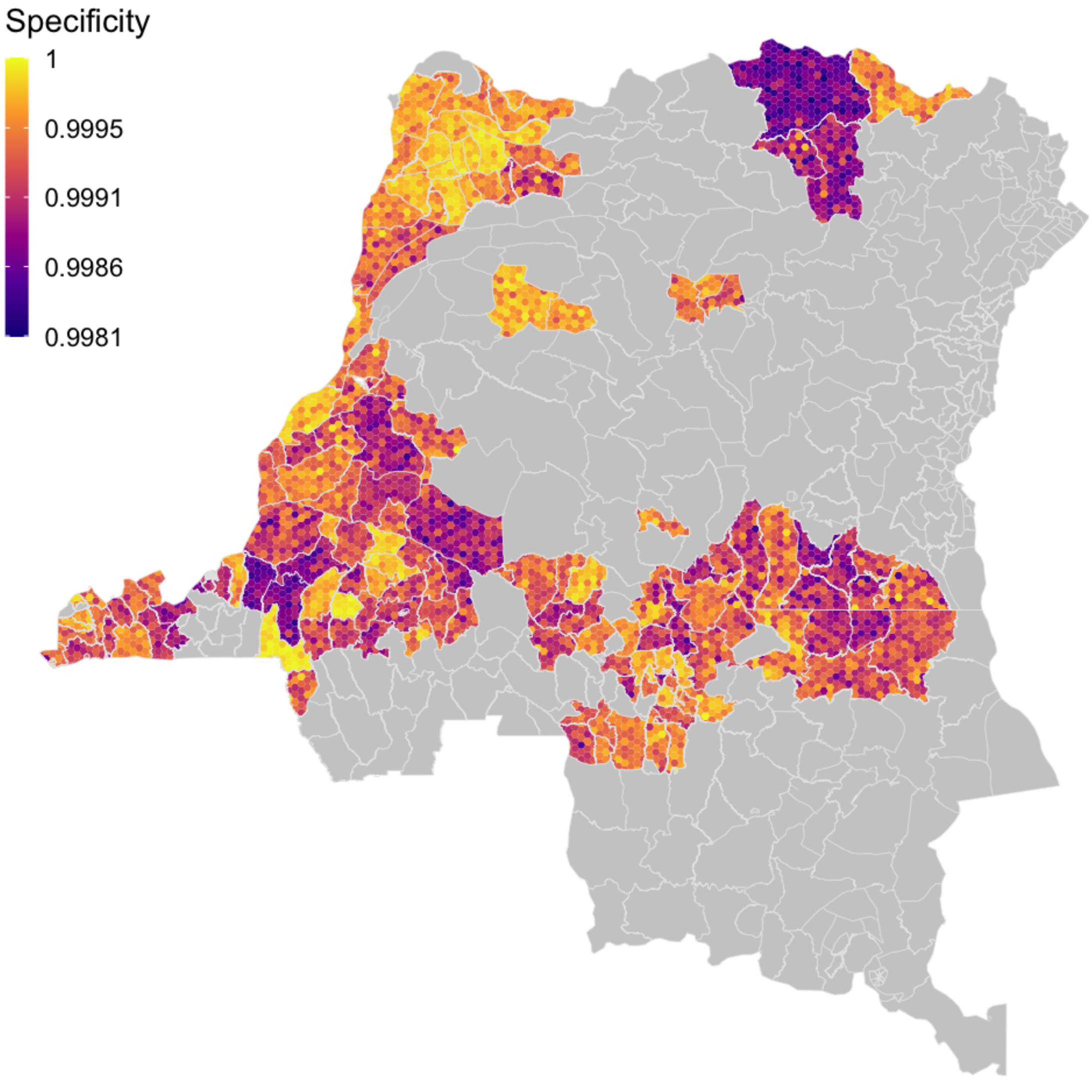

Figure S2.5: Within health zone posterior distribution of the specificity of the diagnostic algorithm, fill colours are a randomly sampled from the posterior distribution for the health zone. Shapefiles used to produce these maps are available under an ODC-ODbL licence at `https://data.humdata.org/dataset/drc-health-data`.

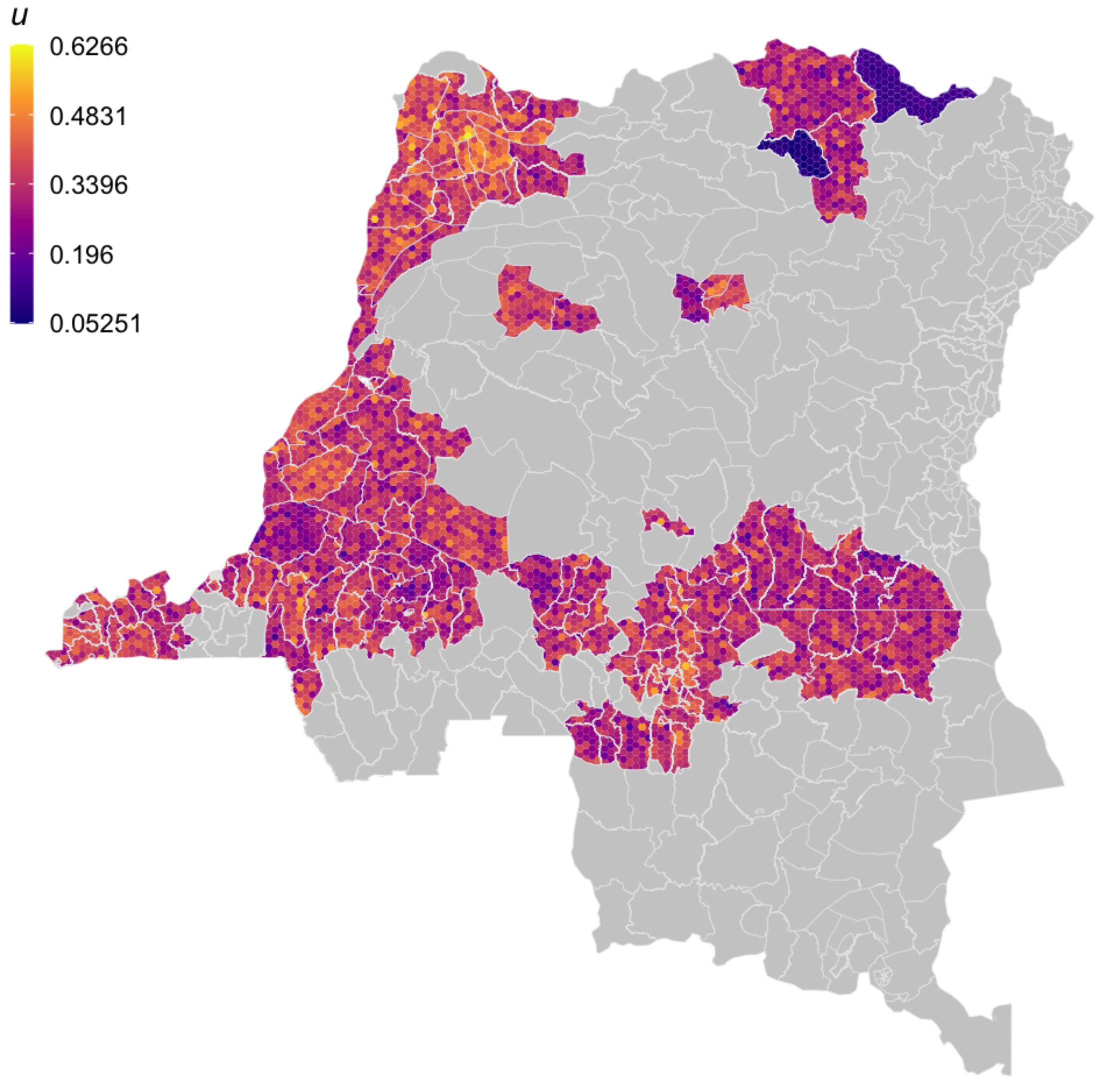

Figure S2.6: Within health zone posterior distribution of u, the proportion of stage 2 passive cases reported. The fill colours are a randomly sampled from the posterior distribution for the health zone. Shapefiles used to produce these maps are available under an ODC-ODbL licence at `https://data.humdata.org/dataset/drc-health-data`.

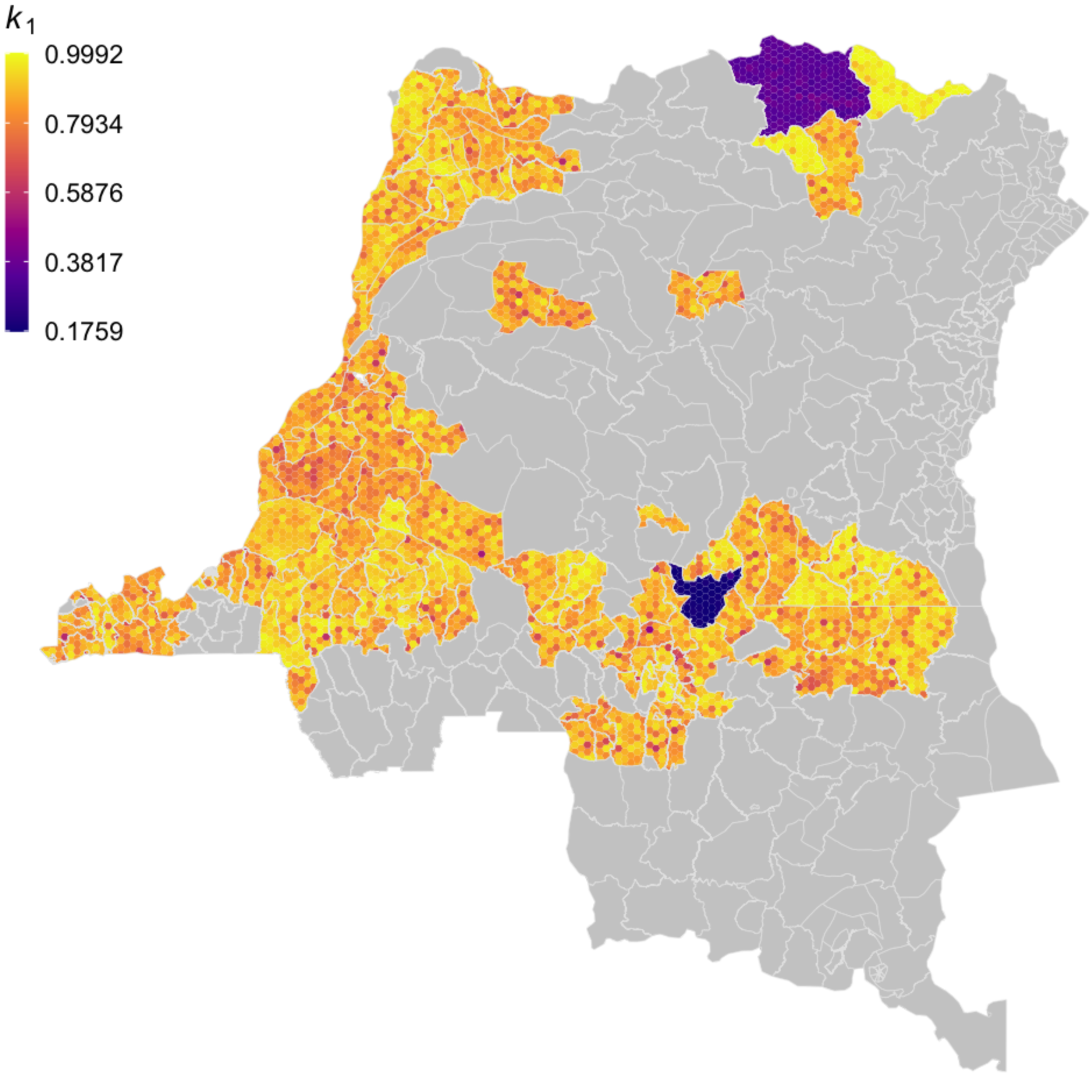

Figure S2.7: Within health zone posterior distribution of k_1, the proportion of low-risk people. The fill colours are randomly sampled from the posterior distribution for the health zone. Shapefiles used to produce these maps are available under an ODC-ODbL licence at `https://data.humdata.org/dataset/drc-health-data`.

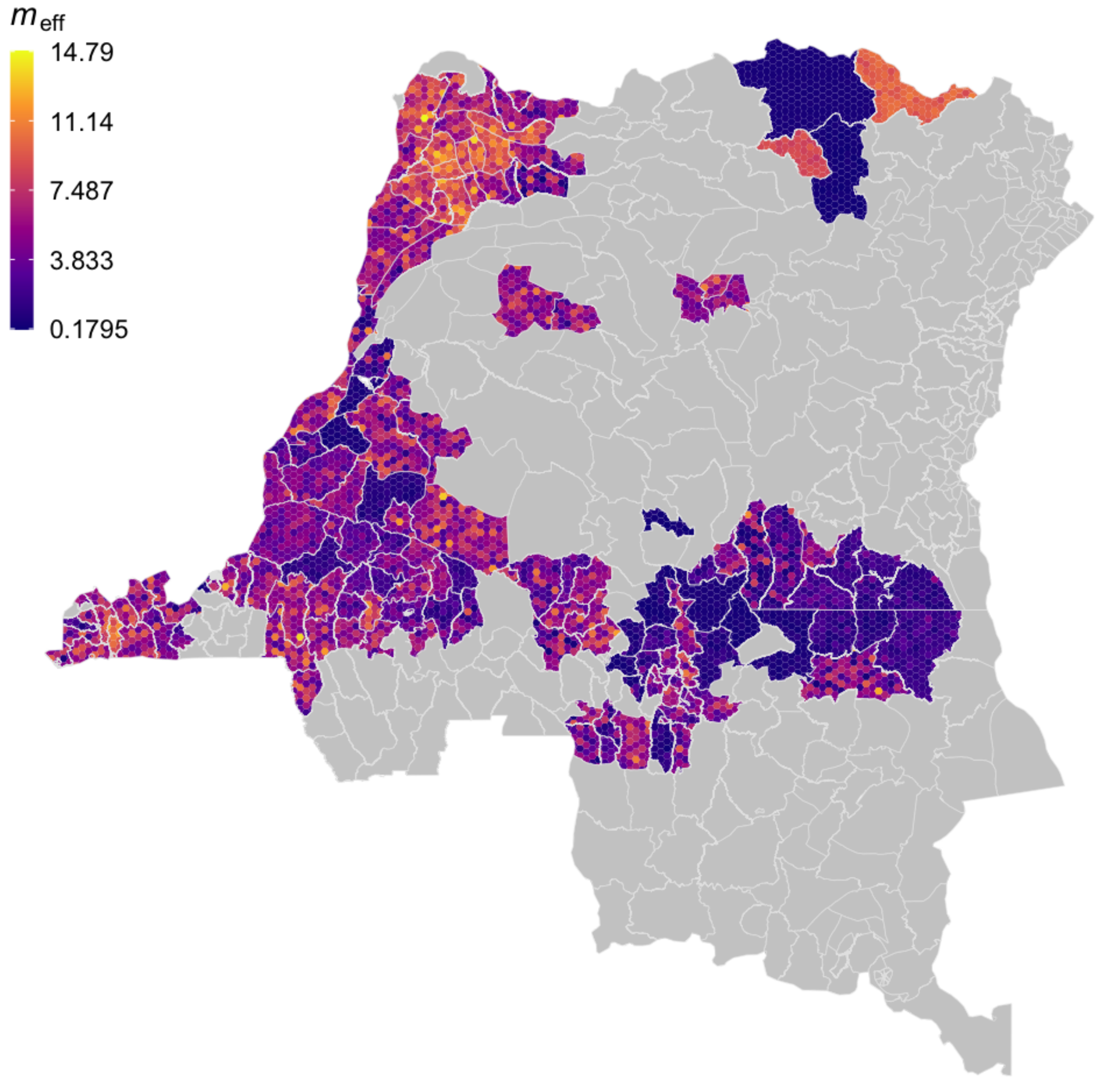

Figure S2.8: Within health zone posterior distribution of m_{eff}; the effective, relative density of tsetse to humans. Fill colours are determined by randomly sampled values from the posterior distribution of m_{eff} from the analysis of the health zone. Shapefiles used to produce these maps are available under an ODC-ODbL licence at `https://data.humdata.org/dataset/drc-health-data`.

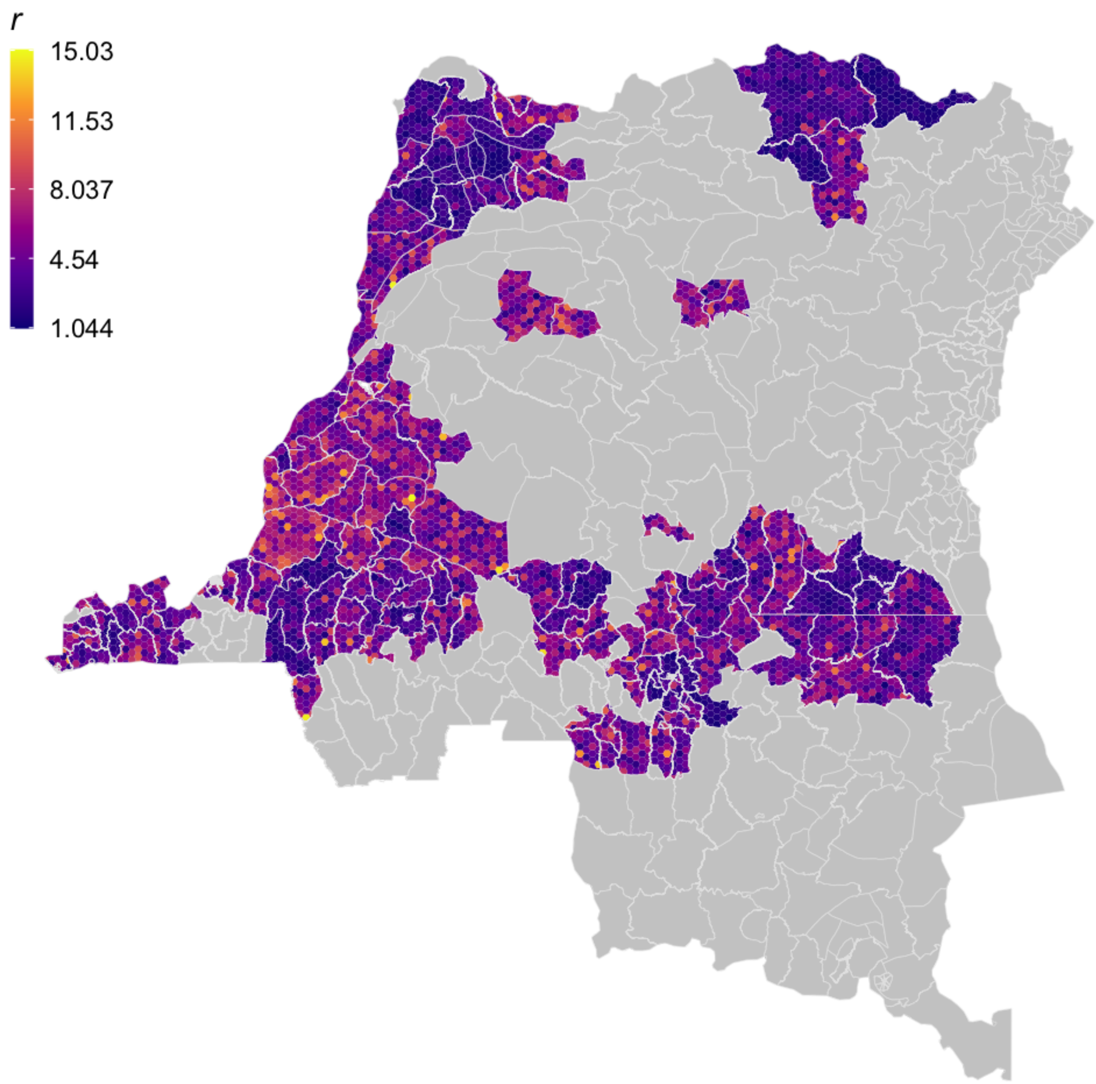

Figure S2.9: Within health zone posterior distribution of r, the relative bites taken on high-risk humans. Fill colours are determined by randomly sampled values from the posterior distribution of r from the analysis of the health zone. Shapefiles used to produce these maps are available under an ODC-ODbL licence at `https://data.humdata.org/dataset/drc-health-data`.

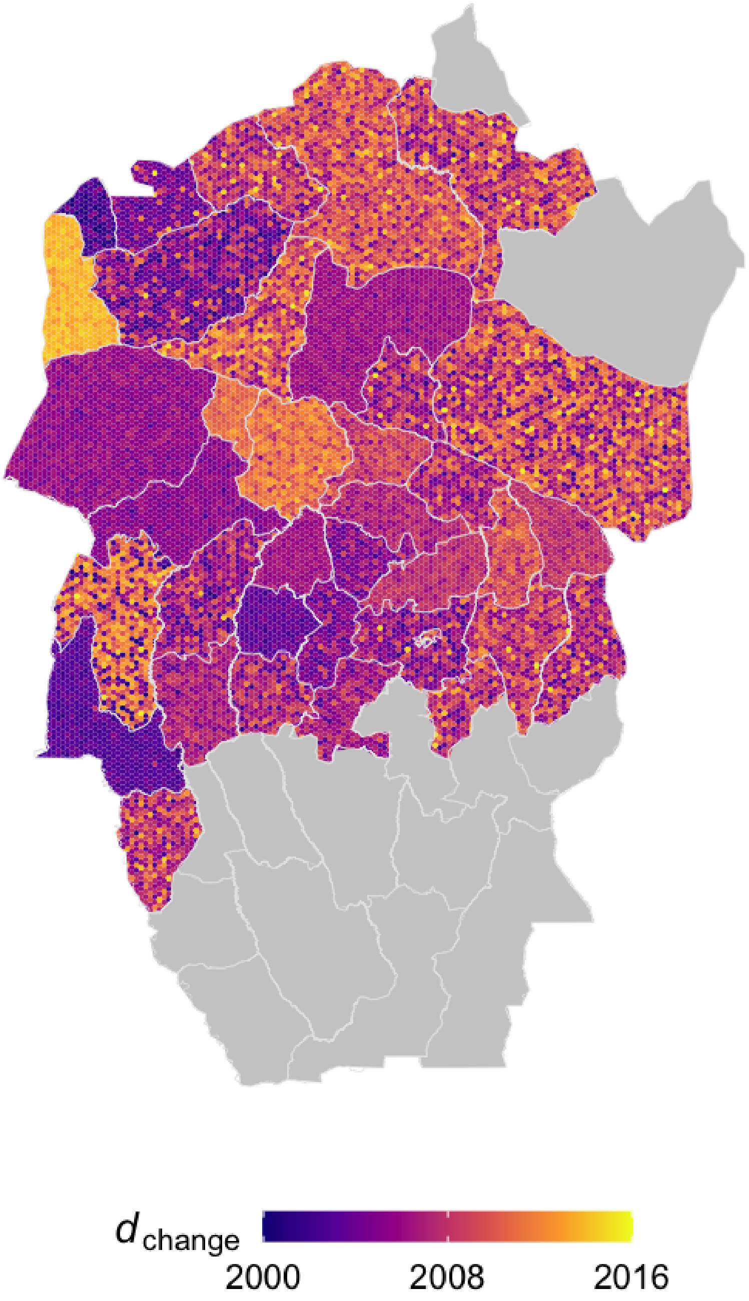

Figure S2.10: Within health zone posterior distribution of d_{change}, the midpoint year for passive improvement, for the former province of Bandundu. Fill colours are randomly sampled from the posterior distribution for the health zone. Shapefiles used to produce these maps are available under an ODC-ODbL licence at `https://data.humdata.org/dataset/drc-health-data`.

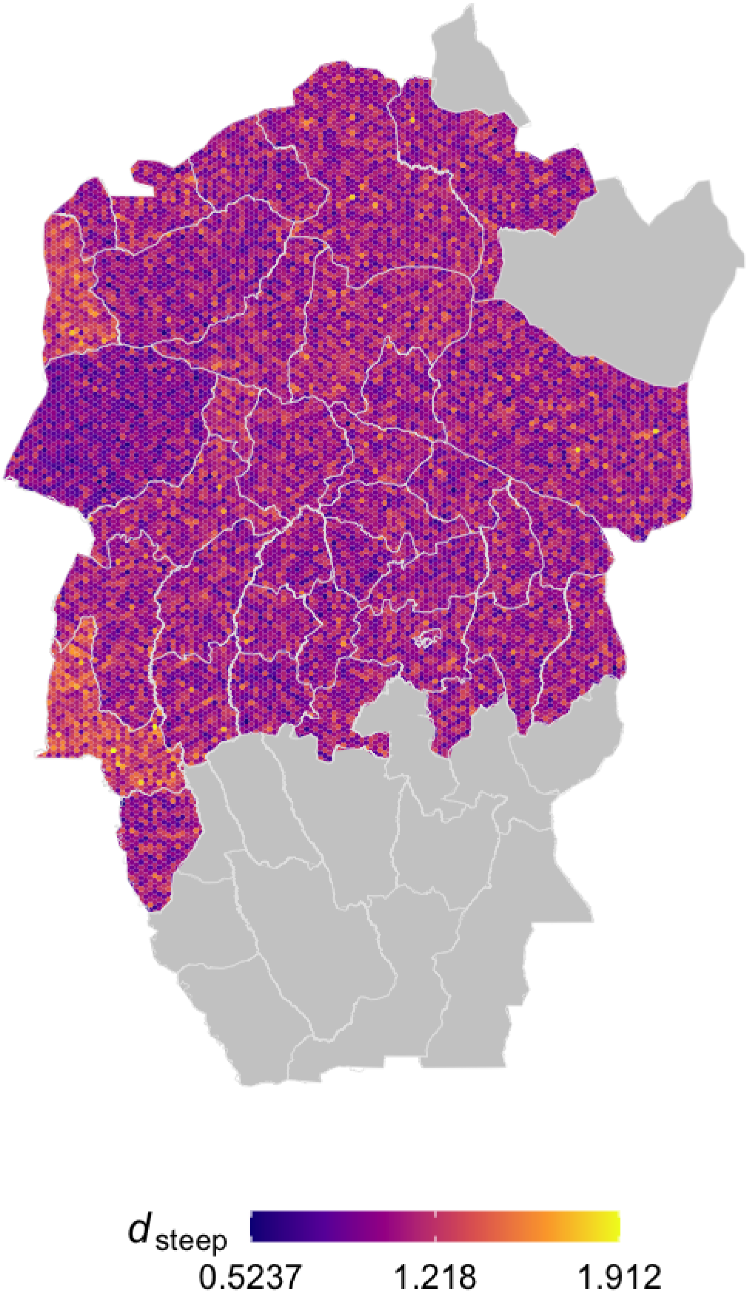

Figure S2.11: Within health zone posterior distribution of d_{steep}, the speed of improvement in passive detection rate, for the former province of Bandundu, fill colours are randomly sampled from the posterior distribution for the health zone. Shapefiles used to produce these maps are available under an ODC-ODbL licence at `https://data.humdata.org/dataset/drc-health-data`.

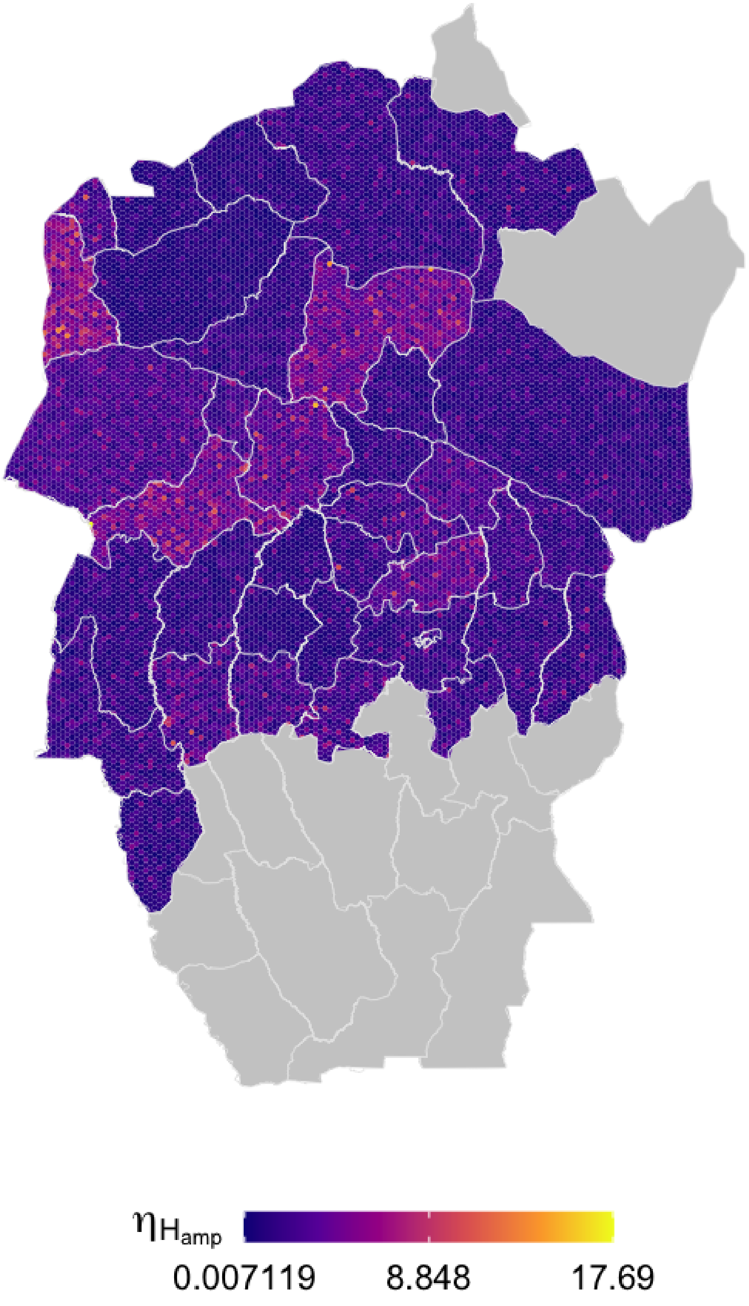

Figure S2.12: Within health zone posterior distribution of $\eta_{H_{amp}}$, the relative improvement in passive stage 1 detection rate, for the former province of Bandundu, fill colours are randomly sampled from the posterior distribution for the health zone. Shapefiles used to produce these maps are available under an ODC-ODbL licence at `https://data.humdata.org/dataset/drc-health-data`.

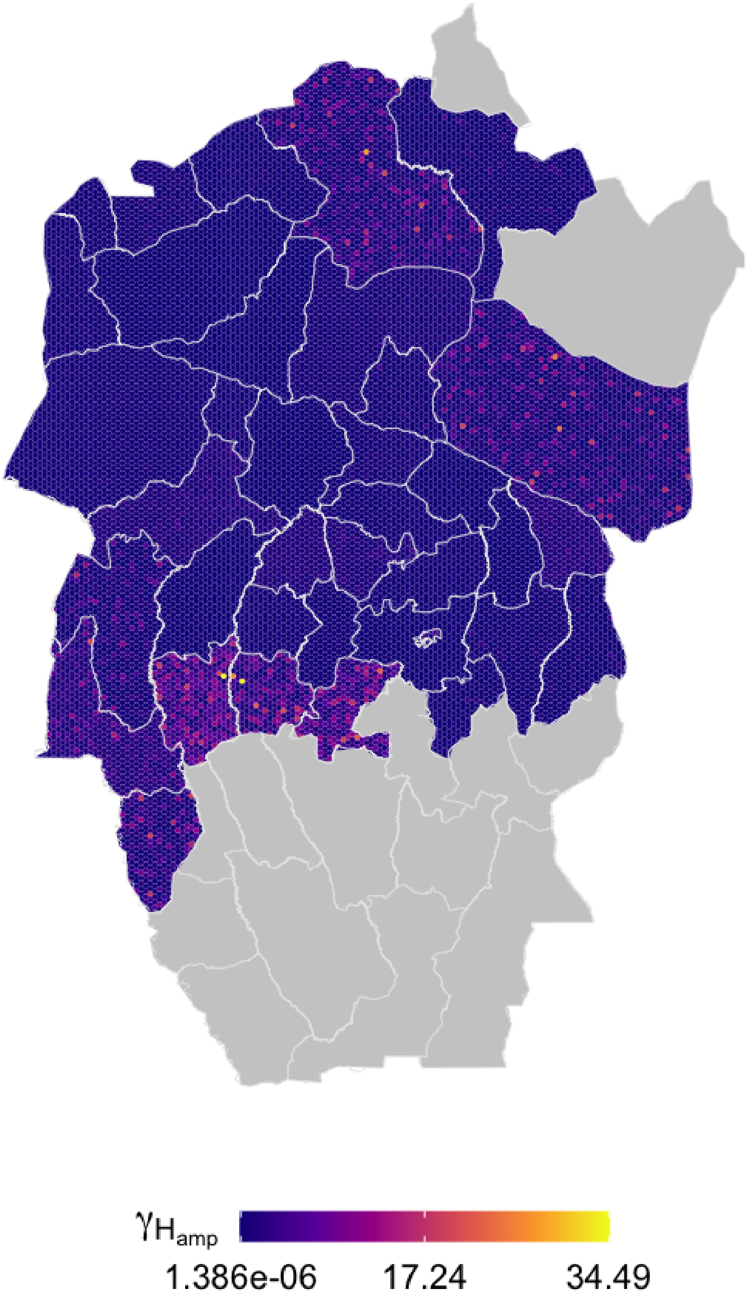

Figure S2.13: Within health zone posterior distribution of $\gamma_{H_{amp}}$, the relative improvement in passive stage 2 detection rate, for the former province of Bandundu, fill colours are randomly sampled from the posterior distribution for the health zone. Shapefiles used to produce these maps are available under an ODC-ODbL licence at `https://data.humdata.org/dataset/drc-health-data`.

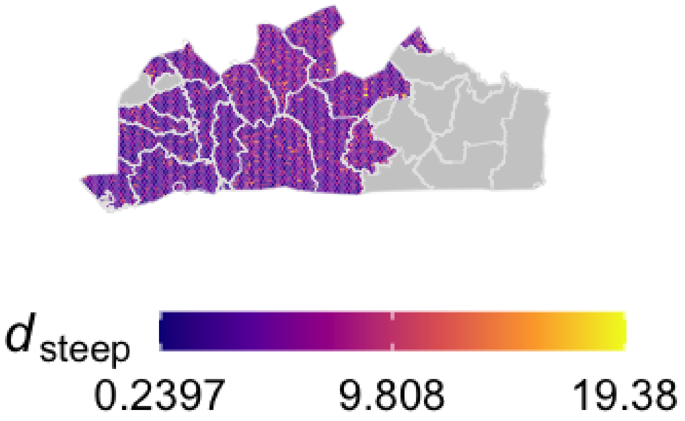

Figure S2.14: Within health zone posterior distribution of d_{steep}, the speed of improvement in passive detection rate, for the former province of Bas Congo, fill colours are randomly sampled from the posterior distribution for the health zone. Shapefiles used to produce these maps are available under an ODC-ODbL licence at `https://data.humdata.org/dataset/drc-health-data`.

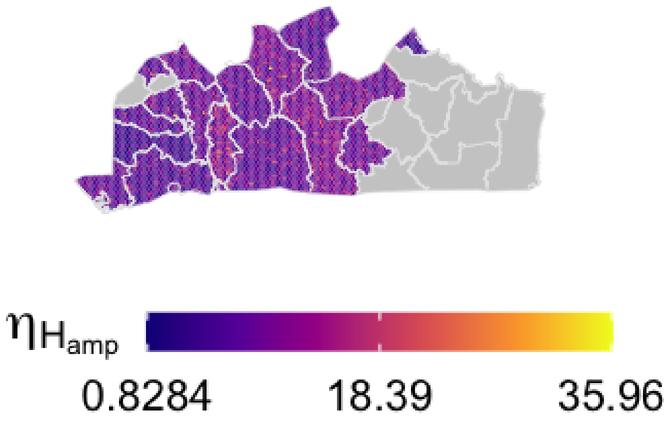

Figure S2.15: Within health zone posterior distribution of $\eta_{\text{H}_{\text{amp}}}$, the relative improvement in passive stage 1 detection rate, for the former province of Bas Congo, fill colours are randomly sampled from the posterior distribution for the health zone. Shapefiles used to produce these maps are available under an ODC-ODbL licence at `https://data.humdata.org/dataset/drc-health-data`.

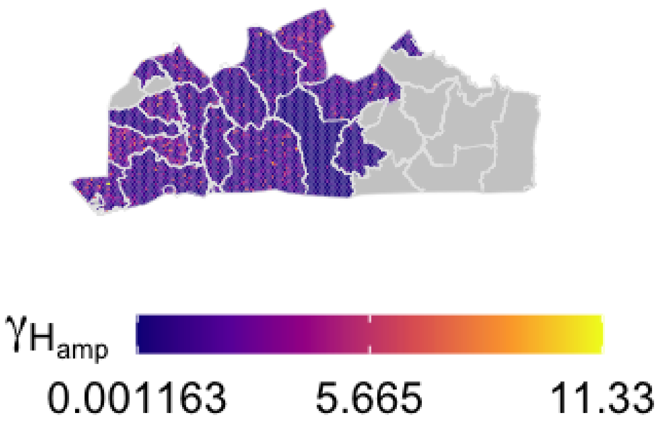

Figure S2.16: Within health zone posterior distribution of $\gamma_{H_{amp}}$, the relative improvement in passive stage 2 detection rate, for the former province of Bas Congo, fill colours are randomly sampled from the posterior distribution for the health zone. Shapefiles used to produce these maps are available under an ODC-ODbL licence at `https://data.humdata.org/dataset/drc-health-data`.
