# Supplementary material for: Quantifying epidemiological drivers of gambiense human African Trypanosomiasis across the Democratic Republic of Congo
Source: PLoS Comput Biol. 2021 Jan 29;17(1):e1008532. doi: 10.1371/journal.pcbi.1008532 (PMC7899378; doi:10.1371/journal.pcbi.1008532)
Supplement: Attachment — Submitted filename: Crump et al Keeling Rock 2020 Epi drivers of Gambian HAT in DRC MS for review PLoS Comp Biol.pdf [file pcbi.1008532.s006.pdf]

PLOS Computational Biology

Quantifying epidemiological drivers of gambiense human African Trypanosomiasis across the Democratic Republic of Congo

--Manuscript Draft--

**Manuscript Number:**	PCOMPBIOL-D-20-01092
**Full Title:**	Quantifying epidemiological drivers of gambiense human African Trypanosomiasis across the Democratic Republic of Congo
**Short Title:**	Epidemiological drivers of gHAT in DRC
**Article Type:**	Research Article
**Keywords:**	gambiense human African trypanosomiasis; deterministic transmission model; adaptive MCMC
**Abstract:**	Gambiense human African trypanosomiasis (gHAT) is a virulent disease declining in burden but still endemic in West and Central Africa. Although it is targeted for elimination of transmission by 2030, there remain numerous questions about the drivers of infection and how these vary geographically.<br>In this study we focus on the Democratic Republic of Congo (DRC), which accounted for 84% of the global case burden in 2016, to explore changes in transmission across the country and elucidate factors which may have contributed to the persistence of disease or success of interventions in different regions. We present a Bayesian fitting methodology, applied to 168 endemic health zones (~100,000 population size), which allows for calibration of mechanistic gHAT model to case data (from the World Health Organization HAT Atlas) in an adaptive and automated framework.<br>It was found that the model needed to capture improvements in passive detection to match observed trends in the data within former Bandundu and Bas Congo provinces indicating these regions have substantially reduced time to detection. Health zones in these provinces generally had longer burn-in periods during fitting due to additional model parameters.<br>Posterior probability distributions were found for a range of fitted parameters in each health zone; these included the basic reproduction number estimates for pre-1998 (R_0) which was inferred to be between 1 and 1.19, in line with previous gHAT estimates, with higher median values typically in health zones with more case reporting in the 2000s.<br>Previously, it was not clear whether a fall in active case finding in the period contributed to the declining case numbers. The modelling here accounts for variable screening and suggests that underlying transmission has also reduced greatly - on average 96% in former Equateur, 93% in former Bas Congo and 89% in former Bandundu - Equateur and Bandundu having had the highest case burdens in 2000. This analysis also sets out a framework to enable future predictions for the country.
**Additional Information:**	
**Question**	**Response**
**Financial Disclosure**<br><br>Enter a financial disclosure statement that describes the sources of funding for the work included in this submission. Review the submission guidelines for detailed requirements. View published research articles from *PLOS Computational Biology* for specific examples.<br><br>This statement is required for submission and **will appear in the published article** if	This work was supported by the Bill and Melinda Gates Foundation (www.gatesfoundation.org) through the Human African Trypanosomiasis Modelling and Economic Predictions for Policy (HAT MEPP) project [OPP1177824] (C.H, R.E.C, P.B, K.S.R. and M.J.K.) and through the NTD Modelling Consortium [OPP1156227, OPP1186851 and OPP1184344] (K.S.R., E.K., S.E.F.S and M.J.K.). The funders had no role in study design, data collection and analysis, decision to publish, or preparation of the manuscript.

the submission is accepted. Please make sure it is accurate.<br><br>**Unfunded studies**<br>Enter: *The author(s) received no specific funding for this work.*<br><br>**Funded studies**<br>Enter a statement with the following details:<br>• Initials of the authors who received each award<br>• Grant numbers awarded to each author<br>• The full name of each funder<br>• URL of each funder website<br>• Did the sponsors or funders play any role in the study design, data collection and analysis, decision to publish, or preparation of the manuscript?<br>• **NO** - Include this sentence at the end of your statement: *The funders had no role in study design, data collection and analysis, decision to publish, or preparation of the manuscript.*<br>• **YES** - Specify the role(s) played.<br><br>* typeset	
**Competing Interests**<br><br>Use the instructions below to enter a competing interest statement for this submission. On behalf of all authors, disclose any competing interests that could be perceived to bias this work—acknowledging all financial support and any other relevant financial or non-financial competing interests.<br><br>This statement **will appear in the published article** if the submission is accepted. Please make sure it is accurate. View published research articles from *PLOS Computational Biology* for specific examples.	The authors have declared that no competing interests exist.

**NO authors have competing interests**<br><br>Enter: *The authors have declared that no competing interests exist.*<br><br>**Authors with competing interests**<br><br>Enter competing interest details beginning with this statement:<br><br>*I have read the journal's policy and the authors of this manuscript have the following competing interests: [insert competing interests here]*<br><br>* typeset	
**Data Availability**<br><br>Authors are required to make all data underlying the findings described fully available, without restriction, and from the time of publication. PLOS allows rare exceptions to address legal and ethical concerns. See the PLOS Data Policy and FAQ for detailed information.<br><br>A Data Availability Statement describing where the data can be found is required at submission. Your answers to this question constitute the Data Availability Statement and **will be published in the article**, if accepted.<br><br>**Important:** Stating 'data available on request from the author' is not sufficient. If your data are only available upon request, select 'No' for the first question and explain your exceptional situation in the text box.<br><br>Do the authors confirm that all data underlying the findings described in their manuscript are fully available without restriction?	No - some restrictions will apply
**Describe where the data may be found in full sentences. If you are copying our sample text, replace any instances of XXX with the appropriate details.**	Data cannot be shared publicly because they were aggregated from the World Health Organisation's HAT Atlas which is under the stewardship of the WHO. Data are available from the WHO (contact J.R. Franco, francoj@who.int) for researchers who meet the criteria for access to confidential data.

Powered by Editorial Manager® and ProduXion Manager® from Aries Systems Corporation

• If the data are **held or will be held in a public repository**, include URLs, accession numbers or DOIs. If this information will only be available after acceptance, indicate this by ticking the box below. For example: *All XXX files are available from the XXX database (accession number(s) XXX, XXX.).*<br>• If the data are all contained **within the manuscript and/or Supporting Information files**, enter the following: *All relevant data are within the manuscript and its Supporting Information files.*<br>• If neither of these applies but you are able to provide **details of access elsewhere**, with or without limitations, please do so. For example:<br><br>*Data cannot be shared publicly because of [XXX]. Data are available from the XXX Institutional Data Access / Ethics Committee (contact via XXX) for researchers who meet the criteria for access to confidential data.*<br><br>*The data underlying the results presented in the study are available from (include the name of the third party and contact information or URL).*<br>• This text is appropriate if the data are owned by a third party and authors do not have permission to share the data.<br><br>* typeset	
Additional data availability information:	

Click here to download Manuscript HAT_MEPP_fitting_Paper.pdf

Quantifying epidemiological drivers of gambiense human African Trypanosomiasis across the Democratic Republic of Congo

Ronald E Crump[1,2,3◑*], Ching-I Huang[1,2◑], Ed Knock[1,4], Simon E F Spencer[1,4], Paul Brown[1,2], Erick Mwamba Miaka[5], Shampa Chancy[5], Matt J Keeling[1,2,3], Kat S Rock[1,2]

1 Zeeman Institute for System Biology and Infectious Disease Epidemiology Research, The University of Warwick, Coventry, U.K.
2 Mathematics Institute, The University of Warwick, Coventry, U.K.
3 The School of Life Sciences, The University of Warwick, Coventry, U.K.
4 The Department of Statistics, The University of Warwick, Coventry, U.K.
5 Programme National de Lutte contre la Trypanosomiase Humaine Africaine (PNLTHA), Kinshasa, D.R.C.

◑These authors contributed equally to this work.
* r.e.crump@warwick.ac.uk

Abstract

Gambiense human African trypanosomiasis (gHAT) is a virulent disease declining in burden but still endemic in West and Central Africa. Although it is targeted for elimination of transmission by 2030, there remain numerous questions about the drivers of infection and how these vary geographically.

In this study we focus on the Democratic Republic of Congo (DRC), which accounted for 84% of the global case burden in 2016, to explore changes in transmission across the country and elucidate factors which may have contributed to the persistence of disease or success of interventions in different regions. We present a Bayesian fitting methodology, applied to 168 endemic health zones (∼100,000 population size), which allows for calibration of mechanistic gHAT model to case data (from the World Health Organization HAT Atlas) in an adaptive and automated framework.

It was found that the model needed to capture improvements in passive detection to match observed trends in the data within former Bandundu and Bas Congo provinces indicating these regions have substantially reduced time to detection. Health zones in these provinces generally had longer burn-in periods during fitting due to additional model parameters.

Posterior probability distributions were found for a range of fitted parameters in each health zone; these included the basic reproduction number estimates for pre-1998 (R_0) which was inferred to be between 1 and 1.19, in line with previous gHAT estimates, with higher median values typically in health zones with more case reporting in the 2000s.

Previously, it was not clear whether a fall in active case finding in the period contributed to the declining case numbers. The modelling here accounts for variable screening and suggests that underlying transmission has also reduced greatly – on average 96% in former Equateur, 93% in former Bas Congo and 89% in former Bandundu – Equateur and Bandundu having had the highest case burdens in 2000. This analysis also sets out a framework to enable future predictions for the country.

Author summary

Gambiense human African trypanosomiasis (gHAT; sleeping sickness) is a deadly disease targeted for elimination by 2030, however there are still several unknowns about what factors influence continued transmission and how this changes with geographic location.

In this study we focus on the Democratic Republic of Congo (DRC), which reported 84% of the global cases in 2016 to try and explain why some regions of the country have had more success than others in bringing down case burden. To achieve this we used a state-of-the-art statistical framework to match a mathematical gHAT model to reported case data for 168 regions with some case reporting during 2000–2016.

The analysis indicates that two former provinces, Bandundu and Bas Congo had substantial improvements to case detection in fixed health facilities in the time period. Overall, all provinces were estimated to have reductions in (unobservable) transmission including ∼96% in former Equateur. This is reassuring as case finding effort has decreased in that region.

The model fitting presented here will allow predictions of gHAT under future strategies to be performed in the future.

Introduction

Gambiense human African trypanosomiasis (gHAT) is a disease caused by the protozoan parasite *Trypanosoma brucei gambiense* which is transmitted by tsetse. The disease has two distinct stages during which the disease progresses from mild to severe, and can lead to death without treatment.

gHAT occurs throughout Western and Central Africa, with 15 countries reporting new cases in the period 2000–2016 [1]. The majority of the detected gHAT cases are in the Democratic Republic of Congo (DRC) where 84% of the new cases in 2016 were reported [2]. While these cases are predominantly in the former province of Bandundu, they are widespread across this large country (230 of 516 health zones had reported cases between 2012 and 2016).

It has long been understood that treatment of gHAT patients not only prevents excess mortality but also can reduce the time spent infectious, and thereby reduce onwards transmission in the population. A combination of active screening (AS) and passive surveillance (PS) followed by treatment of cases has resulted in a decline in the number of new cases from 25,841 (16,951 in DRC) in 2000 to 2,110 (1,768 in DRC) in 2016 [1]. During this time period both diagnostics and drugs for gHAT have evolved with vast improvements for patients.

Traditional AS of at risk populations is done by mobile teams visiting villages and performing an initial mass screen using serological tools (usually the card agglutination test for trypanosomes - CATT), followed by microscopy for serologically positive suspects to confirm presence of the parasite. gHAT treatment in 2016 (and still today) required parasitological confirmation after positive serological testing and before drug administration. For the treatments available in the previous two decades, a final "staging" test – a lumbar puncture to established whether a patient has trypanosomes or elevated white blood cell count in cerebrospinal fluid – was required to select appropriate treatment. This necessary, multi-step diagnostic pathway, currently precludes the possibility of mass drug administration used in control programmes for other NTDs.

For infected people who evade detection by AS due to imperfect diagnostics, non-attendance in AS, or whose village is not screened, it is possible for them to self-present at fixed health facilities and be diagnosed through PS. Not all fixed health facilities have gHAT diagnostics but this has been improved over the last 20 years. Globally, WHO estimate that in 2012, 41%, 71%, and 83% of at-risk population lived

within 1, 3 and 5 hours of health facilities with these diagnostics respectively [3], and by 2017 this increased to 58%, 79%, 89% [1]. It is not clear what quantitative impact this improvement has had on time to detection.

Along with many other neglected tropical diseases (NTDs), gHAT is the subject of two World Health Organisation goals; for gHAT these are (1) elimination as a public health problem by 2020 and (2) elimination of transmission (EOT) by 2030. If we are to truly strive to reach this second elimination of transmission goal, then it is of utmost importance to understand and quantify the reasons for success to date (indeed, it is expected that we are on track for the first goal [1] with some countries identified as eligible for validation of gHAT elimination as a public health problem in the latest WHO report [4]), and identify what factors may have hindered progress.

gHAT transmission is known to be highly focal - now burden of disease is decreasing globally, there remain pockets of infection in geographically disconnected areas [1]. Previously mathematical modelling work has shown that, whilst persistence of infection at very low prevalences is generally surprising for infectious diseases, the timescale of gHAT dynamics enable this infection to remain extant for long periods of time in small settlements [5]. Other transmission modelling has examined drivers of gHAT in specific foci and concluded that, by fitting to longitudinal human case data, there must exist heterogeneity in risk of humans populations both in terms of exposure to tsetse and also in participation in active screening [6, 7]. Other factors which are likely to vary geographically include access to fixed health facilities with gHAT diagnostics - this can impact the time individuals spend infected and the risk that they die without diagnosis - and the regional density of tsetse. At present it is, however, unknown which drivers are influencing transmission in different regions.

In the present study we consider what epidemiological variables are driving transmission across the health zones ($\sim$ 100,000 population size) of DRC using a dynamic transmission model, and examine how control interventions from 2000 to 2016 have impacted infection and modified some of these variables over time. To achieve parameter estimation across the country we utilise an automated Bayesian fitting procedure with an adaptive Metropolis-Hastings random walk and in-built convergence diagnostics. The samples from the posterior probability distributions of fitted parameters from this method can be used to examine within health zone parameter averages and uncertainty as well as comparing estimates across health zones. The mechanistic model can be used to infer the level of transmission to humans over time even though it is not directly observable in data; the posterior parameters are used to do this.

Materials and methods

Data

Data on gHAT cases in DRC were obtained from the WHO who curate the global HAT Atlas database [1, 8, 9]. The data in the HAT Atlas are aggregated case data by location (defined by the available geolocation and geographical identifier information), year and surveillance type (active or passive screening). There were 117,573 records in the HAT Atlas data file covering the period 2000–2016, although very little staging information was available prior to 2015.

Because administrative areas may be redefined or renamed over time, the HAT Atlas records were matched to a single, recent map obtained as a shapefile (UCLA, personal communication). Matching to the shapefile was performed by geolocation, where available, plus an identifier matching both to geolocated locations and directly to the administrative areas stored in the shapefile. Other geographical data were sourced from

the Humanitarian Data Exchange – an older United Nations Office for the Coordination of Humanitarian Affairs (OCHA) health zone shapefile, and geolocations of localities (OCHA) and health facilities (Global Healthsite Mapping Project) within DRC. The additional geographical data provided alternative names for administrative/health areas as well as names and geolocations of locations and hence facilitate the data cleaning and matching process. While geolocations (longitude and latitude) were available for most records they may not be reliable indicators of position as they may have been assigned as the centroid of the lowest available level administrative/health area if not recorded directly. Area and location names from all sources were sanitised before use in matching. Sequential matching was then carried out, with unmatched records being carried forward to the next step. The location and health site lists were combined and located on the UCLA shapefile to obtain administrative area names consistent with this map. Detail on this process is available in the Supplementary Information.

Following data matching, the data were aggregated within health zone and year to produce health zone level data sets for all health zones in which cases were reported. It is noted that for a small number of health zone–year combinations the reported number of cases detected by active screening was more than the declared number of people screened, which was zero in some instances.

Cases of gHAT are not recorded across all of the DRC; for many regions it is because they have not historically observed gHAT cases and are not believed to be endemic for gHAT, but for others it is due to challenging accessibility. Figure 1 shows the regions where there were no data as well as the status of the analysis performed in the present study. Inference was not performed in all health zones where there were data available; either because there were too few observations (Data < 10), no cases were detected (No Detections) or transmission is not believed to be taking place due to lack of tsetse habitat (No Transmission). Inference was deemed to be possible where there were 10 or more observations, where an observation was an aggregate record within year relating to either more than 20 people being actively screened or cases detected passively.

Figure 2 contains three maps of aggregated numbers of new cases by health zone for three non-consecutive five-year periods spanning the period 2000–2016 for which data were available. These maps illustrate the decrease in cases reported over time, in particular in the former provinces of Equateur (in the North-West of DRC), which had the highest burden in 2000, and Bandundu (situated to the east of Kinshasa), which despite its decrease is now the most highly-endemic region globally. In the North there was an increase in reported cases due to the presence of Médecins Sans Frontières (MSF) who use an alternative diagnostic algorithm to that used by the national programme. The MSF algorithm did not feature parasitological confirmation after serological testing, and hence had a higher sensitivity but lower specificity (similar to those described in [10]).

Model

gHAT infection model

The gHAT model equations are given below (1) and correspond with Fig 3. The model is a variant "Model 4" of that presented elsewhere [6, 7, 11–14].

In the present study human hosts are assumed to be either at low-risk and randomly participate in screening (subscript $H1$), or high-risk and never participate in screening (subscript $H4$). Tsetse bites are assumed to be taken on humans or non-reservoir animals. However, the non-reservoir animal species do not need to be explicitly modelled.

The model is parameterised with a combination of fixed and fitted parameters. Fixed parameters (see Table 1) generally correspond to assumed biological values which

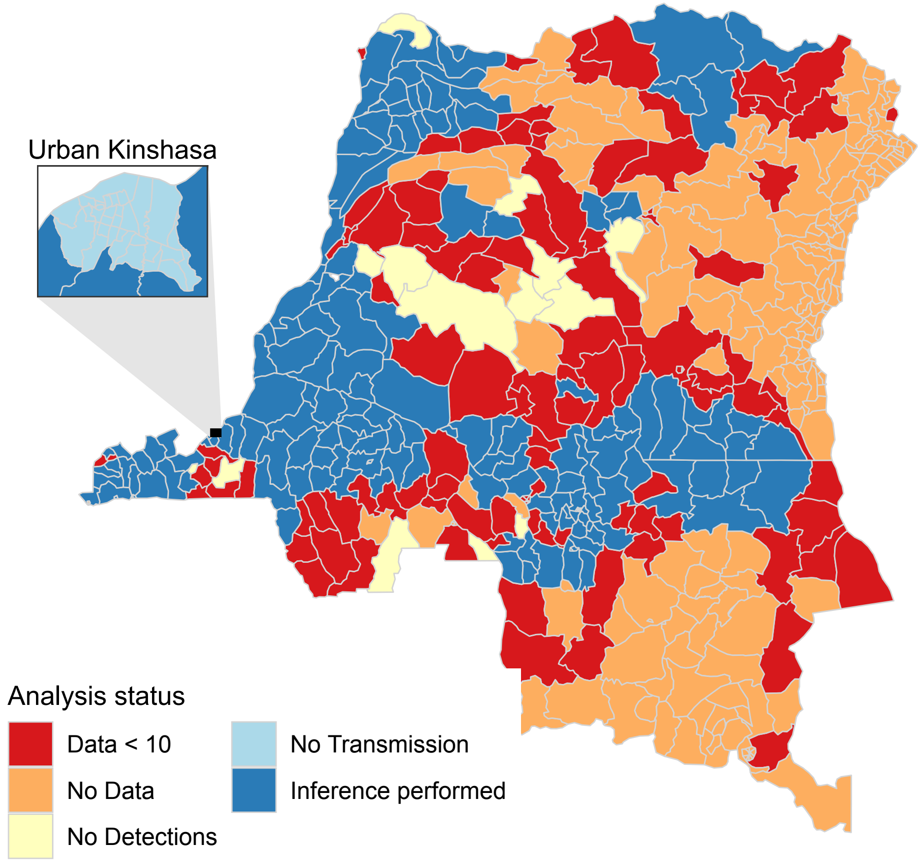

Fig 1. Status of the individual health zone-level analyses, if the analysis was not run the reason is indicated.

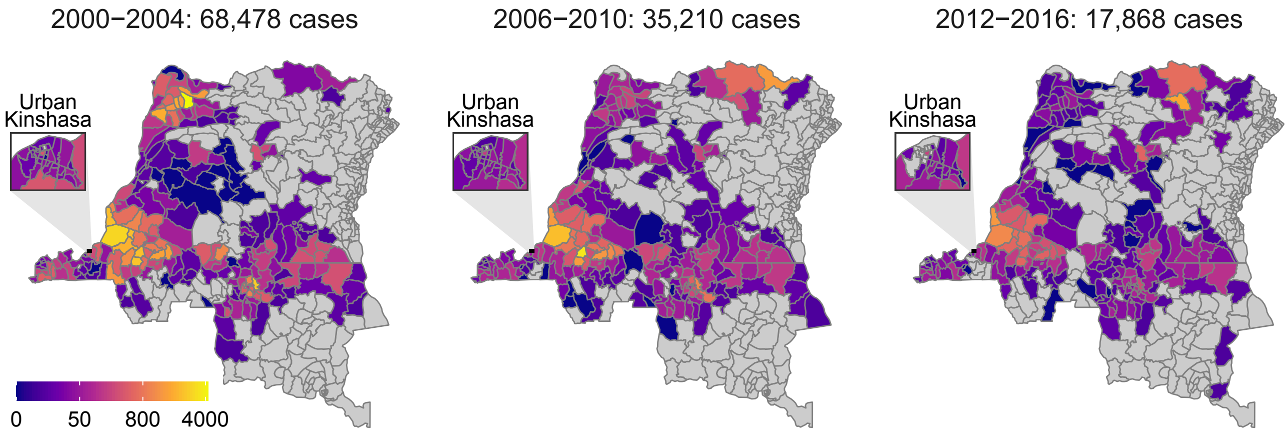

Fig 2. Total number of new cases recorded within health zone and five-year period.

are unlikely to vary across the DRC such as the human mortality rate, tsetse bite rate and stage 1 to stage 2 disease progression in humans. Fitted parameters (see Table 2)

are those which are likely to be correlated with region including the tsetse-to-human relative density, the proportion of the population who are at high-risk of exposure to tsetse, the reporting rate (linked to access to health facilities with gHAT testing capacity), and parameters linked to the time to passive detection.

Table 1. Model parameterisation (fixed parameters). Notation, a brief description, and the values used for fixed parameters.

Notation	Description	Value	
N_H	Total human population size in 2015	Fixed for each health zone	[15]
μ_H	Natural human mortality rate	5.4795×10^{-5} days^{-1}	[16]
B_H	Total human birth rate	$=\mu_H N_H$	
σ_H	Human incubation rate	0.0833 days^{-1}	[17]
φ_H	Stage 1 to 2 progression rate	0.0019 days^{-1}	[18, 19]
ω_H	Recovery rate or waning-immunity rate	0.006 days^{-1}	[20]
Sens	Active screening diagnostic sensitivity	0.91	[10]
B_V	Tsetse birth rate	0.0505 days^{-1}	[21]
ξ_V	Pupal death rate	0.037 days $^{-1}$	
K	Pupal carrying capacity	$=111.09N_H$	[21]
$\mathbb{P}$(pupating)	Probability of pupating	0.75	
μ_V	Tsetse mortality rate	0.03 days^{-1}	[17]
σ_V	Tsetse incubation rate	0.034 days^{-1}	[22, 23]
α	Tsetse bite rate	0.333 days^{-1}	[24]
p_V	Probability of tsetse infection per single infective bite	0.065	[17]
ε	Reduced non-teneral susceptibility factor	0.05	[25]
f_H	Proportion of blood-meals on humans	0.09	[26]
disp_{act}	Overdispersion parameter for active detection	4×10^{-4}	–
$\text{disp}_{\text{pass}}$	Overdispersion parameter for passive detection	2.8×10^{-5}	–

Value of B_V is chosen to maintain constant population size without interventions. Value of K is chosen to reflect the observed bounce back rate.

Table 2. Model parameterisation (fitted parameters). Notation, brief description, and information on the prior distributions for fitted parameters.

**Notation**	**Description**	**Prior distribution**[1]	**Percentiles of prior distribution** [2.5, 50 & 97.5%]	**Unit**
R_0	Basic reproduction number (NGM approach)	$X \sim 1 + \mathrm{Exp}(10)$	[1.003, 1.069, 1.369]	-
r	Relative bites taken on high-risk humans	$X \sim 1 + \Gamma(3.68, 1.09)$	[2.015, 4.654, 10.028]	-
k_1	Proportion of low-risk people	$X \sim \mathrm{B}(16.97, 3.23)$	[0.6564, 0.8514, 0.9609]	-
k_4	Proportion of high-risk people		$k_4 = 1 - k_1$	-
η_H^{post}[2]	Treatment rate from stage 1, 1998 onwards	$X \sim \Gamma\big(3.54, 5.32 \times 10^{-5}\big)$	[4.59, 17.1, 42.9]$\times 10^{-5}$	days^{-1}
γ_H^{post}[2]	Treatment rate from stage 2, 1998 onwards	$X \sim \Gamma(2.45, 0.00192)$	[7.59, 40.7, 121]$\times 10^{-4}$	days^{-1}
$b_{\gamma_H^{\mathrm{pre}}}$	Relative treatment rate from stage 2 factor, pre-1998	$X \sim \mathrm{B}(1, 1)$	[0.025, 0.500, 0.975]	-
γ_H^{pre}	Treatment rate from stage 2, pre-1998		$\gamma_H^{\mathrm{pre}} = b_{\gamma_H^{\mathrm{pre}}} \gamma_H^{\mathrm{post}}$	days^{-1}
Spec	Active screening diagnostic specificity	$X \sim 0.998 + (1 - 0.998)\,\mathrm{B}(7.23, 2.41)$	[0.9989, 0.9995, 0.9999]	-
u	Proportion of stage 2 passive cases reported	$X \sim \mathrm{B}(20, 40)$	[0.2208, 0.3315, 0.4564]	-
d_{change}[3]	Midpoint year for passive improvement	$X \sim 2000 + (2017 - 2000)\,\mathrm{B}(5, 6)$	[2003.2, 2007.7, 2012.5]	Year
$\eta_{H_{\mathrm{amp}}}$[4]	Relative improvement in passive stage 1 detection rate	$X \sim \Gamma(2.013, 1.049)$	[0.258, 1.775, 5.870]	-
$\gamma_{H_{\mathrm{amp}}}$[4]	Relative improvement in passive stage 2 detection rate	$X \sim \Gamma(1.001, 5)$	[0.127, 3.471, 18.455]	-
d_{steep}[4]	Speed of improvement in passive detection rate	$X \sim \Gamma(39.57, 0.0270)$	[0.761, 1.058, 1.424]	years^{-1}

[1]Where Exp(.), Γ(.) and B(.) are the exponential, gamma (parameterised with shape and scale) and beta distributions, respectively. [2]Former province-specific priors used for η_H^{post} and γ_H^{post}; prior distributions and percentiles for Bandundu presented, see SI for other former provinces. [3]d_{change} is only fitted in the former province of Bandundu. [4]$\eta_{H_{\mathrm{amp}}}$, $\gamma_{H_{\mathrm{amp}}}$ and d_{steep} are only fitted in the former provinces of Bandundu and Bas Congo; the prior distributions and percentiles presented relate to Bandundu, see SI for Bas Congo.

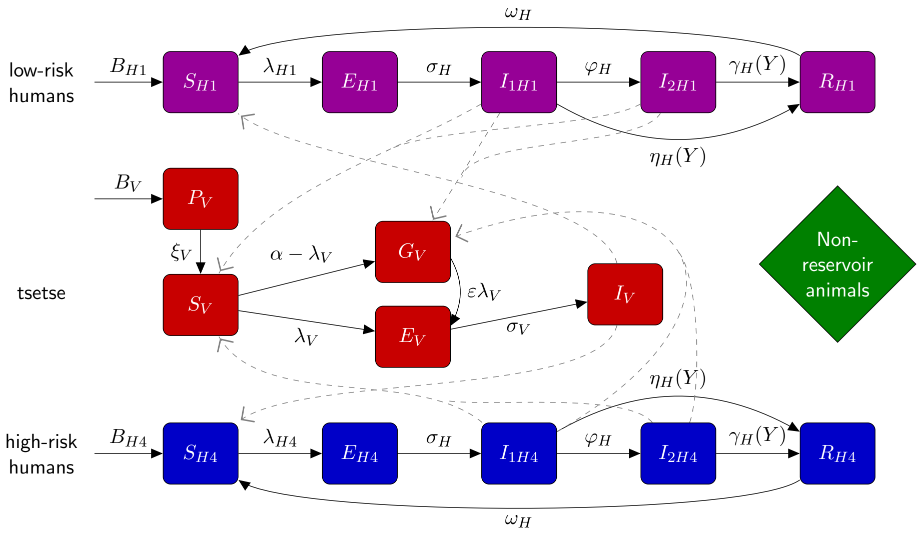

Fig 3. Illustration of compartmental HAT model. Multi-host model of HAT with one host species able to confer HAT (humans), a further non-reservoir species (others) and tsetse. After a short incubation period, infected human hosts follow the progression which includes an infectious stage I disease, I_{1H}, infectious stage II disease, I_{2H}, and a non-infectious (due to hospitalisation) disease, R. Pupal stage tsetse, P_V, emerge into unfed adults. Unfed tsetse are susceptible, S_V, and following a blood-meal become either exposed, E_V, or have reduce susceptibility to the trypanosomes, G_V. Tsetse select their blood-meal from one of the host types dependant upon innate feeding preference and relative host abundance. High-risk humans are r-fold more likely to receive bites than low-risk humans. Any blood-meals taken upon "other" hosts do not result in infection. The transmission of infection between humans and tsetse is shown by grey paths. This figure is adapted from the original model schematic [6].

$$
\text{Humans}\left\{
\begin{aligned}
\frac{\mathrm{d}S_{Hi}}{\mathrm{d}t} &= \mu_H N_{Hi} + \omega_H R_{Hi} - \alpha m_{\mathrm{eff}} f_i \frac{S_{Hi}}{N_{Hi}} I_V - \mu_H S_{Hi}\\
\frac{\mathrm{d}E_{Hi}}{\mathrm{d}t} &= \alpha m_{\mathrm{eff}} f_i \frac{S_{Hi}}{N_{Hi}} I_V - (\sigma_H + \mu_H) E_{Hi}\\
\frac{\mathrm{d}I_{1Hi}}{\mathrm{d}t} &= \sigma_H E_{Hi} - (\varphi_H + \eta_H(Y) + \mu_H) I_{1Hi}\\
\frac{\mathrm{d}I_{2Hi}}{\mathrm{d}t} &= \varphi_H I_{1Hi} - (\gamma_H(Y) + \mu_H) I_{2Hi}\\
\frac{\mathrm{d}R_{Hi}}{\mathrm{d}t} &= \eta_H(Y) I_{1Hi} + \gamma_H(Y) I_{2Hi} - (\omega_H + \mu_H) R_{Hi}
\end{aligned}
\right.
$$

$$
\text{Tsetse}\left\{
\begin{aligned}
\frac{\mathrm{d}P_V}{\mathrm{d}t} &= B_V N_H - (\xi_V + \tfrac{P_V}{K}) P_V\\
\frac{\mathrm{d}S_V}{\mathrm{d}t} &= \xi_V \mathbb{P}(\text{survive pupal stage}) P_V - \alpha S_V - \mu_V S_V\\
\frac{\mathrm{d}E_{1V}}{\mathrm{d}t} &= \alpha\big(1 - f_T(t)\big) p_V \big(\textstyle\sum_i f_i \frac{(I_{1Hi} + I_{2Hi})}{N_{Hi}} + f_A \frac{I_A}{N_A}\big)(S_V + \varepsilon G_V)\\
&\qquad - (3\sigma_V + \mu_V + \alpha f_T(t)) E_{1V}\\
\frac{\mathrm{d}E_{2V}}{\mathrm{d}t} &= 3\sigma_V E_{1V} - (3\sigma_V + \mu_V + \alpha f_T(t)) E_{2V}\\
\frac{\mathrm{d}E_{3V}}{\mathrm{d}t} &= 3\sigma_V E_{2V} - (3\sigma_V + \mu_V + \alpha f_T(t)) E_{3V}\\
\frac{\mathrm{d}I_V}{\mathrm{d}t} &= 3\sigma_V E_{3V} - (\mu_V + \alpha f_T(t)) I_V\\
\frac{\mathrm{d}G_V}{\mathrm{d}t} &= \alpha(1 - f_T(t)) S_V\\
&\qquad -\alpha\Big(f_T(t) + (1 - f_T(t)) p_V \varepsilon \big(\textstyle\sum_i f_i \frac{(I_{1Hi} + I_{2Hi})}{N_{Hi}} + f_A \frac{I_A}{N_A}\big) G_V\Big)\\
&\qquad\quad - \mu_V G_V
\end{aligned}
\right.
\tag{1}
$$

The actual number of vectors is $S_V, E_{1V}, E_{2V}, E_{3V}, I_V$ and G_V multiplied by N_V/N_H, where N_V is the total population of adult tsetse and $N_H = N_{H1} + N_{H4}$ denotes the total human population. Then, the effective probability of human infection per single infective tsetse bite m_{eff} is defined as $N_V p_H / N_H$ with the original vector-to-human transmission probability p_H.

The proportion of tsetse bites taken on low-risk and high-risk humans are f_1 and f_4, depending on the relative availability/attractiveness and the relative abundance of two risk groups. High-risk humans are assumed to be r-fold more likely to receive bites, i.e. $s_1 = 1$ and $s_4 = r$. Therefore, f_i's can be calculated using $f_i = \dfrac{s_i N_{Hi}}{\sum_j s_j N_{Hj}}$.

Improvements to passive case detection

For simulations in this study, we assumed that prior to 1998 there was limited passive case detection, which would not detect stage 1 cases ($\eta_H^{\mathrm{pre}} = 0$) and have a slower time to detection for stage 2 ($\gamma_H^{\mathrm{pre}} = b_{\gamma_H^{\mathrm{pre}}} \times \gamma_H^{\mathrm{post}}$ where $b_{\gamma_H^{\mathrm{pre}}} = [0, 1]$). In 1998 we assume that the introduction of the card agglutination test for trypanosomes (CATT) enabled better diagnosis and stage 1 and 2 rates were increased to η_H^{post} and γ_H^{post} respectively.

As explored in a previous modelling study focusing on former Bandundu province [14], there is strong evidence of improvements to the passive surveillance

system during 2000–2012 from examining the changes to the proportion of passive cases identified in stage 1 compared to stage 2. The health-zone level data utilised here for the main model fitting do not contain staging information before 2015 (and generally there are relatively few cases in 2015 and 2016). The staged case information from 2015 and 2016 were aggregated to the former province level and used alongside the provincial-level staged case data for 2000–2012 [27] to set prior distributions for logistic functions which we use to describe improvements to both the passive stage 1 and stage 2 detection rates in each year:

$$\eta_H(Y) = \eta_H^{\text{post}} \left[1 + \frac{\eta_{H_{\text{amp}}}}{1 + \exp\Big(-d_{\text{steep}}(Y - d_{\text{change}})\Big)} \right] \tag{2}$$

$$\gamma_H(Y) = \gamma_H^{\text{post}} \left[1 + \frac{\gamma_{H_{\text{amp}}}}{1 + \exp\Big(-d_{\text{steep}}(Y - d_{\text{change}})\Big)} \right] \tag{3}$$

These functional forms were used in former Bandundu and Bas Congo provinces where there was strong evidence of improvement in the data. In Bas Congo the "change year", d_{change}, was fixed to 2015.5 as this corresponds to the year in which there was a substantial increase in the number of fixed health facilities with gHAT rapid diagnostic tests (RDTs) [28].

It is noted that only the stage 1 detection rate was changed in Model W in [14] as this provided a good fit to the data at the province level. Here we alter both stage 1 and stage 2, firstly because it is logical that passive detection improvements would lead to faster rates for both stages of disease, and secondly as this is better able to capture passive detection patterns observed in health zone level data. In the present study other provinces were assumed to have constant passive detection rates since 1998.

Active screening algorithms

Active screening since 2000 has generally comprised of mobile teams screening as many people as possible in villages using a multi-diagnostic algorithm. The first diagnostic used is the CATT test on finger prick blood, and this may be followed by CATT dilutions and finally microscopy to visually confirm presence of the parasite. After parasite confirmation, the individual is a confirmed case and required further testing using lumbar puncture to diagnose disease stage to be able to provide the correct stage-specific treatment. In the model we assume this algorithm has a sensitivity of 0.91, and very high but imperfect specificity with a prior around 0.9995 and fitted to health zone level data.

Whilst the national programme, PNLTHA, have consistently used the algorithm with parasitilogical confirmation, in former Oriental province in the North of the country, some of the screening activities (pre-2013) were performed by MSF. The MSF algorithm was more contracted and individuals who were found positive on a CATT 1:32 were reported as cases and given treatment. Therefore, a higher fixed sensitivity (MSF sensitivity = 0.95 in contrast to PNLTHA sensitivity = 0.91) and a lower fitted specificity (= $b_{\text{specificity}} \times$ specificity with targeted mean = 0.991, where $b_{\text{specificity}} = [0, 1]$) are used for years up to 2012 in these regions.

Since 2015, video confirmation of parasitological diagnosis was introduced to Mosango and Yasa Bonga health zones in former Bandundu province. This additional validation diagnostic is designed to ensure quality control of case confirmation, which is especially important as there become very few cases remaining. The model uses the assumption that there have therefore been no false positives in active screening since 2015 in Mosango and Yasa Bonga.

Vector control

Between 2000–2016 there were very limited vector control activities in DRC, with tsetse control implemented in a single health zone, Yasa Bonga in former Bandundu province, via the deployment of tiny targets since mid-2015. This method of control, using insecticidal impregnanted blue targets, has successfully reduced fly populations in other countries (90% in Uganda [29], 80% in Guinea, and 99% in Chad). In the present study we incorporate vector control with a 90% reduction in tsetse for Yasa Bonga [30] (see SI for further details).

Likelihood

Eight parameters; $R_0, r, \eta_H^{\text{post}}, \gamma_H^{\text{post}}, b_{\gamma_H^{\text{pre}}}, k_1, u$, and Spec were fitted in all health zones. Additional parameters were included as required (combinations of d_{change}, $\eta_{H_{\text{amp}}}$, $\gamma_{H_{\text{amp}}}$, d_{steep} and $b_{\text{specificity}}$ as appropriate, see above).

The Metropolis-Hastings MCMC used a log-likelihood function:

$$\begin{aligned}
LL(\theta|x) = {} & \log(P(x|\theta)) \\
\propto & \sum_{i=2000}^{2016} \Bigg(\log \left[\text{BetaBin} \left(A_{D1}(i) + A_{D2}(i); z(i), \frac{A_{M1}(i) + A_{M2}(i)}{z(i)}, \text{disp}_{\text{act}} \right) \right] \\
& + \log \left[\text{Bin} \left(A_{D1}(i); A_{D1}(i) + A_{D2}(i), \frac{A_{M1}(i)}{A_{M1}(i) + A_{M2}(i)} \right) \right] \\
& + \log \left[\text{BetaBin} \left(P_{D1}(i) + P_{D2}(i); N_H, \frac{P_{M1}(i) + P_{M2}(i)}{N_H}, \text{disp}_{\text{pass}} \right) \right] \\
& + \log \left[\text{Bin} \left(P_{D1}(i); P_{D1}(i) + P_{D2}(i), \frac{P_{M1}(i)}{P_{M1}(i) + P_{M2}(i)} \right) \right] \Bigg)
\end{aligned}$$

The model takes parameterisation θ, x is the data, $P_{Dj}(i)$ and $A_{Dj}(i)$ are the number of passive/active cases (of stage j) in year i of the data, $P_{Mj}(i)$ and $A_{Mj}(i)$ are the number of passive/active cases (of stage j) in year i of the model, and $z(i)$ is the number of people screened in year i. $\text{BetaBin}(m; n, p, \rho)$ gives the probability of obtaining m successes out of n trials with probability p and overdispersion parameter ρ. The overdispersion accounts for larger variance than under the binomial. The pdf of this distribution is given by:

$$\text{BetaBin}(m; n, p, \rho) = \frac{\Gamma(n+1)\Gamma(m+a)\Gamma(n-m+b)\Gamma(a+b)}{\Gamma(n-m+1)\Gamma(n+a+b)\Gamma(a)\Gamma(b)}$$

where $a = p(1/\rho - 1)$ and $b = a(1-p)/p$.

Consequently larger ρ yield more overdispersion. To avoid overfitting, the overdispersion parameters were manually tuned to be appropriate for a health zone level fit, and thereafter left fixed across MCMC runs (Table 1).

For four health zones there was no screening reported in one or two years in which there was more than 25 active cases recorded. In this scenario the number of negative test results in year i, $A_D^-(i)$, was fitted as a negative binomially distributed variable and $\hat{z}(i) = A_{D1}(i) + A_{D2}(i) + A_D^-(i)$ was used in the calculation of the log-likelihood.

Health zone results are aggregated by provinces and are compared to the province level data as a check that the health zone fits make sense.

Priors

Prior distributions for the fitted parameters are given in Table 2 for the parameters fitted in all health zones and those fitted in former Bandundu province. Information on

other former province–specific priors is given in the SI.

Informative priors were used for most parameters. For the parameters relating to treatment rates from stages 1 and 2 (η_H^{post} and γ_H^{post}), and improvement in passive detection ($\eta_{H_{\text{amp}}}$, $\gamma_{H_{\text{amp}}}$ and d_{steep}) the priors were based on posterior distributions of these parameters from fitting the model to data at the former–province level.

Markov chain Monte Carlo algorithm

For each health zone the model was fitted using the adaptive Metropolis-Hastings random walk algorithm [31]. Two independent chains were run in parallel from different starting values. The chains were run in 3 phases: a transient phase, an adaptive phase and a sampling phase and only samples from the final phase were used in the analysis.

The aim of the transient phase was to move the chains towards the posterior mass. In this phase, lasting $B = 500$ iterations, single site parameter updates were used with proposal standard deviation σ_n^i for parameter i in proposal n. If proposal n was accepted then $\sigma_{n+1}^i = 2\sigma_n^i$ and if it was rejected then $\sigma_{n+1}^i = 2^{a/(a-1)}\sigma_n^i$. Here, we target an acceptance rate of $a = 0.44$, which was found to be optimal in [32].

The aim of the adaptive phase was to begin learning the covariance matrix of the posterior in order to find an efficient proposal. In this phase proposals were drawn from $\boldsymbol{Y}_{n+1} \sim N_d(\boldsymbol{X}_n, \frac{2.38^2\lambda_n^2}{d}\boldsymbol{\Sigma}_n)$, where $\boldsymbol{X}_n$ is the location of the chain after n iterations. The covariance matrix was initially $\boldsymbol{\Sigma}_B$ (the initial variances used can be found in the SI) and subsequently

$$\boldsymbol{\Sigma}_{n+1} = \frac{n-B}{n-B+10}\text{Cov}(\boldsymbol{X}_{B+1},\ldots,\boldsymbol{X}_n) + \frac{10}{n-B+10}\boldsymbol{\Sigma}_B.$$

The scaling factor was $\lambda_n = 1$ on even iterations and updated adaptively on odd iterations, namely $\lambda_{n+2} = x_n\lambda_n$ if iteration n was accepted and $\lambda_{n+2} = x_n^{a/(a-1)}\lambda_n$ if rejected. We used $x_n = 1 + \frac{50}{50+n-B}$ and targeted an acceptance rate of $a = 23.4\%$, which was found to be optimal in [32].

The duration of the last two phases was determined adaptively, by examining the Gelman-Rubin convergence diagnostic [33] and the Effective Sample Size (ESS) diagnostic [34]. The adaptive phase was increased iteratively by 100 up to a maximum of 10^5 iterations until the following convergence criteria were satisfied for the most recent 2000 observations. First $R_{\text{within}}^{(i,j)} < 1.1$ for every parameter i and chain j; and second $R_{\text{between}}^{(i)} < 1.5$ for all i.

Finally, in the sampling phase, proposals were drawn as for the adaptive phase. The duration of this phase was determined based on ESS. To achieve a sample of 2000 states with an ESS of at least 1000, each chain was run for 1000 iterations, thinned by a factor $\kappa = 1$ and the ESS calculated to be twice the minimum ESS over the parameters and the two chains. If the ESS criterion was not satisfied then a further 1000 iterations were sampled from each chain and the thinning factor κ was increased by 1, up to a maximum of 200.

Results

Fig 4 shows the maximum values of the Gelman-Rubin convergence diagnostic for each health zone level analysis within former province. A good level of convergence was achieved, with only 5 of 168 analyses having $R^{(i)} > 1.02$ while 140 of the 168 analyses had $R^{(i)} \leq 1.01$.

The maximum number of iterations for the adaptive phase was required in 29 analyses. Of these, 18 were in the former province of Bandundu in which all health zone

analyses included fitting of additional parameters to account for changes in the effectiveness of passive surveillance. It may be possible to improve the adaptive MCMC to reduce the number of analyses carrying out the maximum adaptive phase length, however the levels of convergence already achieved imply that the effort required may not result in markedly improved analysis results.

The observed effective sample size (ESS) exceeded 1,000 in 140 of the individual health zones analyses, and was less than 500 for 13 analyses. As with the adaptive phase, 14 of 28 analyses with an ESS less than 1,000 were for health zones in Bandundu. This again reflects the additional complexity of fitting the model in this former province.

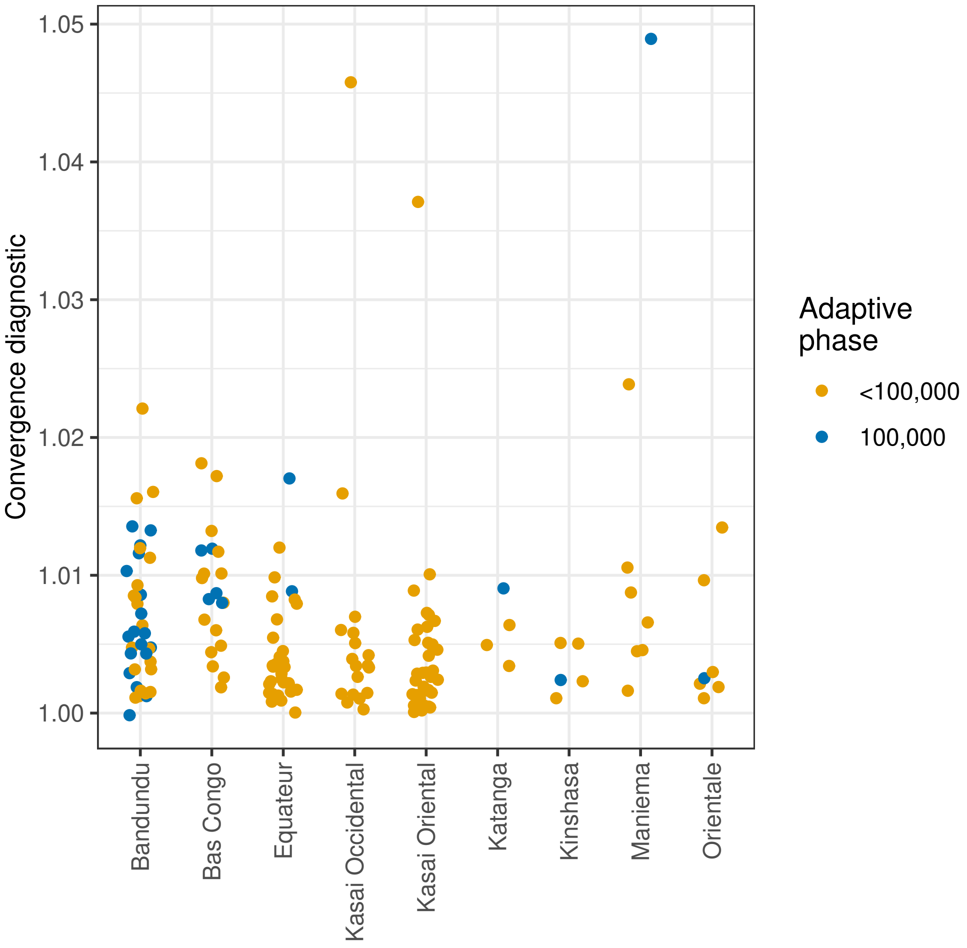

Fig 4. Maximum Gelman-Rubin convergence diagnostics grouped by former province and whether or not the maximum length of adaptive phase (100,000 iterations) was reached.

The fitting process matches model outcomes to reported timeseries of actively- and passively-detected cases. Fig 5 shows examples of these trends for two example health zones; Kwamouth (in the former province of Bandundu) and Tandala (in the former province of Equateur). Kwamouth has a much higher incidence of gHAT infection than Tandala, and consequently has higher numbers of people being actively screened each year – the average number of people actively screened annually in Kwamouth was 52% of the estimated population in 2015 (127,205), while this was 6.8% for Tandala

(estimated 2015 population of 274,945). Fig 5 shows how well the model fits to the timeseries of reported cases both actively and passively detected. In addition, Fig 5 also shows the unobservable downward trend in new infections estimated by the model. The results of all 168 health zone level fits including inferences about annual numbers of new infections, are available online (S3 Online Results).

In Kwamouth health zone there is a noticeable "humped" trend in passive case detection, with higher case reporting in 2005–2008. The model is able to replace this trend by increasing the passive detection rates, $\eta_H(Y)$ and $\gamma_H(Y)$, and importantly we infer that underlying transmission actually declines during this time period, despite increasing reporting (see Figure 5 and S3 Online Results). This humped shape of passive case reporting is observed in many health zones of former Bandundu province, such as Bokoro, Bolobo, Ipamu, and Yasa Bonga. Much of former Equateur province has another typical shape passive detection trend which looks similar to exponential decay especially in the north (e.g. Bominenge, Boto, Budjala, Gemena, Karawa, Kungu, Libenge, Tandala) which had very high case reporting in early 2000s. In these locations we reproduce the trend using fixed passive case detection combined with impactful active screening.

Table 3 contains former province-level estimates of the number of new infections in 2000, 2008 and 2016 and the percentage reduction in new infections between these years. The values from each of 1,000 posterior samples for each analysed health zone within a former province were summed to give 1,000 former province–level values, each health zone being independent in our analyses. Over this period, a 96% median reduction in the number of new infections has been achieved in Equateur. The largest percentage reductions in the number of new infections are seen in the former provinces with the highest reported cases in 2000. Notably the province with the highest reporting in 2000 (Equateur), was not estimated to have highest transmission (Bandundu was higher), however Equateur does have a huge inferred reduction in transmission of 96%. Despite a calculated 89% transmission reduction, Bandundu province remained the province with most ongoing transmission in 2016.

Table 3. Reduction in new gHAT infections by former province. Medians and 95% credible intervals (CIs) of aggregated health zone-level outcomes.

Former province	New infections (median [95% CI])			Percentage reduction	
	2000	2008	2016	2000–2008	2000–2016
Bandundu	7344 [6733, 7977]	3187 [2931, 3466]	808 [684, 950]	57 [55, 58]	89 [87, 90]
Bas Congo	804 [723, 889]	204 [181, 230]	58 [45, 76]	75 [72, 77]	93 [90, 95]
Equateur	4565 [4152, 4980]	446 [404, 490]	161 [136, 189]	90 [89, 91]	96 [96, 97]
Kasai Occidental	701 [601, 825]	312 [270, 362]	163 [133, 198]	55 [51, 60]	77 [71, 81]
Kasai Oriental	4184 [3672, 4740]	2284 [1769, 2840]	1892 [1261, 2583]	45 [39, 53]	55 [44, 66]
Katanga	145 [109, 193]	135 [102, 178]	117 [85, 161]	7 [-3, 17]	20 [-1, 37]
Kinshasa	161 [126, 226]	96 [76, 126]	66 [46, 94]	41 [26, 52]	59 [39, 74]
Maniema	233 [189, 292]	174 [144, 214]	150 [118, 194]	25 [16, 35]	35 [20, 50]
Orientale	1936 [1701, 2194]	1336 [1130, 1547]	672 [531, 833]	31 [22, 40]	65 [58, 72]

The fits of the model to the reported case timeseries as exemplified in Fig 5 reflect the parameters of the model, many of which are estimated within the fitting process. Fig 6 illustrates the posterior distribution of R_0 within each health zone for which inference was performed. The map of DRC was partitioned into hexagons and partial hexagons, based on health zone boundaries, which were then filled with a colour based on a random value from the posterior distribution of R_0 for that health zone. By representing the posterior distributions in this way the aim is to illustrate how both the level and variability of the parameter differs between health zones. Further examples of

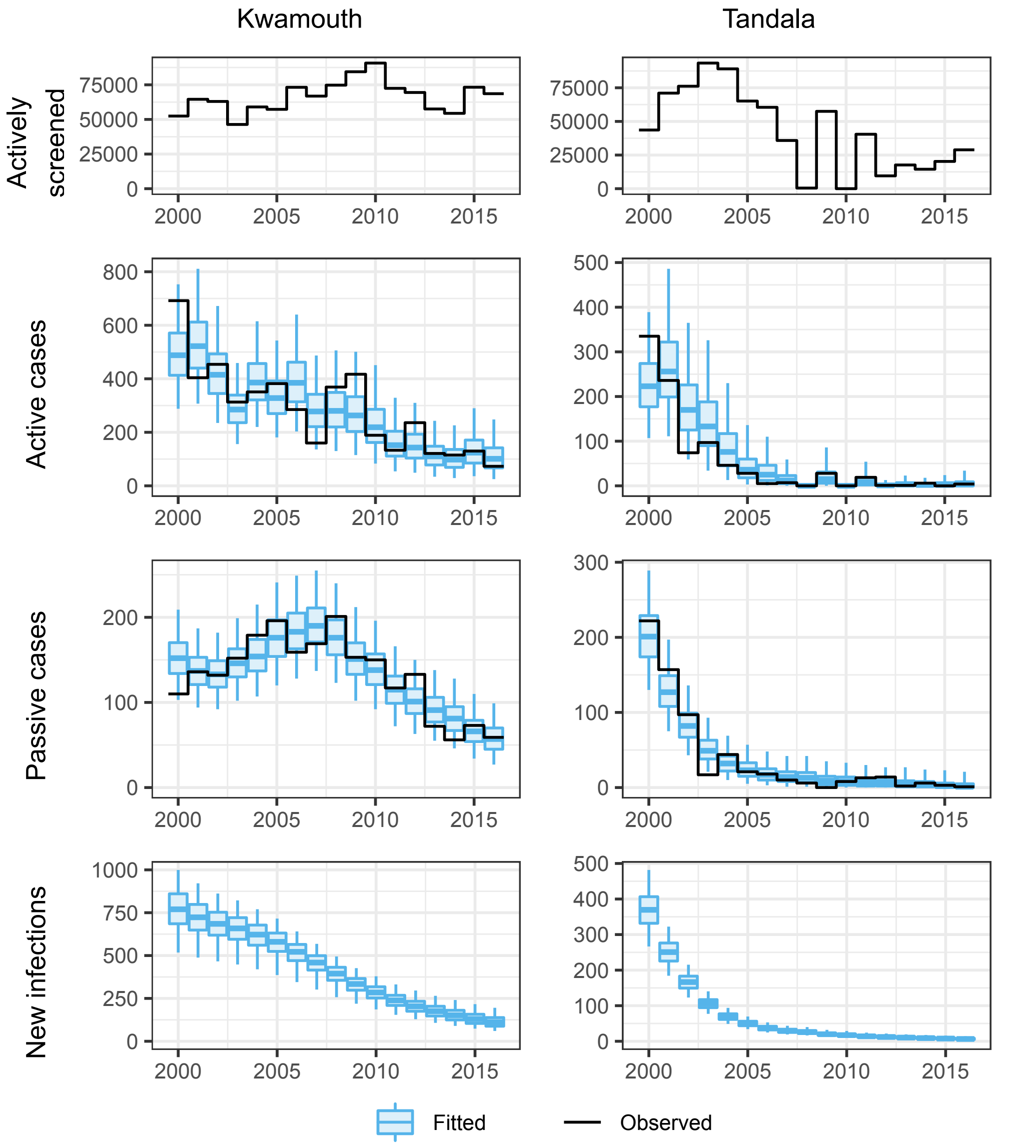

Fig 5. Demonstration of fit to the observed trends in new case detection over time and predicted numbers of new infections for two example health zones; Kwamouth in former Bandundu province and Tandala in former Equateur province.

this representation of posterior distributions are available for the parameters fitted across all health zone analyses in S2 Supplementary Figures and online (S3 Online Results).

The R_0 posterior maps shows that some of the highest R_0 estimates are in Kwamouth health zone, former Bandundu province, although more generally R_0 estimates remain very low - typically only slightly above one - for the whole country. R_0 is a bundled parameter, linked to the ability of an infection to persist for a particular geographic setting. Higher R_0 values on the map roughly correspond to health zones which have reported high number of cases historically.

Maps showing posterior passive detection rates (see η_H^{post} in SI) show that there is geographic variability in time to detection, with typically quicker diagnosis in 2000 in

former Equateur province and slower times in former Orientale, Maniema and Katanga. The average time spent infectious if not identified by active screening can be estimated in each health zone using the posterior parameters (see SI Section S1.3); in Kwamouth health zone this duration changes from a median of 1153 days in 2000 to 784 days in 2016 and in Tandala this is fixed at 716 days between 2000 and 2016.

Active diagnostic algorithm specificity is computed to be very high, with former Equateur province having near perfect specificity.

The parameter u corresponds with the proportion of cases that are reported if not picked up in active screening. High u is interpreted as high reporting and fewer deaths outside the health care system. Average reporting generally follows the prior of u although it appears that former Equateur has better reporting (lower underreporting/deaths). The estimated proportion of reporting including active screening can be computed using model outputs for cases and deaths (see SI Section S1.4). In Kwamouth this is estimated to have changed from 0.6 (95%: 0.53–0.82) in 2000 to 0.81 (95%: 0.62–0.93) in 2016, while in Tandala it fluctuated over time from 0.65 (95%: 0.53–0.75) in 2000 and finishing back at 0.65 (95%: 0.0–1.0) in 2016, with the median varying between 0.40 (in 2010) and 0.76 (in 2003). The low case numbers in later years are why there is a higher uncertainty in percentage reporting in 2016.

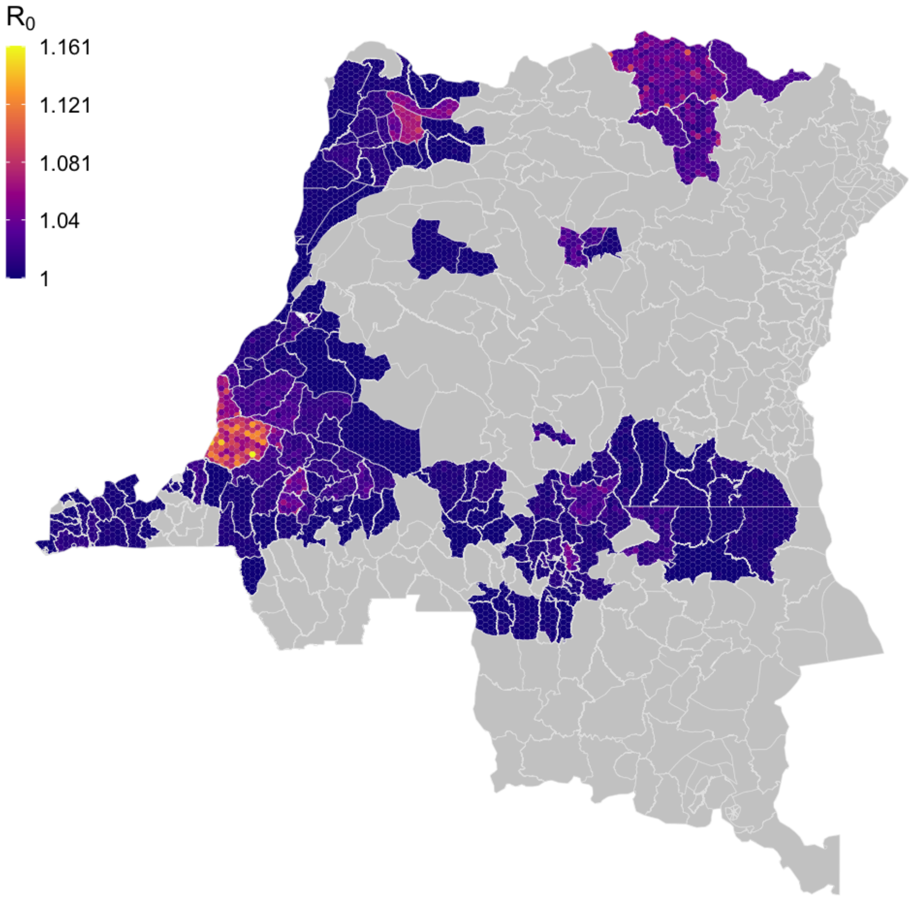

Fig 6. Within health zone posterior distribution of R_0. Fill colours for hexagons within a health zone are determined by randomly sampled values from the posterior distribution of R_0 from the analysis of that health zone.

Discussion

In this study the fitting of a transmission model for gHAT has been automated allowing health zone-level model fitting across the whole of DRC to be performed for the first time. This fitting process enables exploration of the underlying epidemiology of gHAT at the health zone level, estimating parameters of the transmission model based on historical data.

The fits highlight the success of past interventions, both in the obvious decline in the number of reported cases but also through quantification of improvements in surveillance, such as in the case of changes in passive surveillance over time in Bandundu and Bas Congo.

Even in areas where there has been a decline in active screening activity over time (especially in former Equateur province where the mean annual screening from 2012–2016 was ∼150,000 compared to a maximum in 2003 of around 900,000), the modelling indicates that there has been a real reduction in transmission (∼96% in Equateur), rather than simply a decline in reported cases due to scaling back the detection effort.

The model used here did not consider the presence of possible animal reservoirs or transmission via asymptomatic humans. Historically, gHAT has been generally regarded as an anthroponosis [35], lacking an animal reservoir which would hinder or prevent elimination of the disease. More recently there have been concerns that this may not be the case; *T.b. gambiense* has been identified in various animals, and have been shown to be transmissible to tsetse experimentally [36]. Mathematical modelling has attempted to ascertain likelihood of animal transmission through various model comparison exercises [6, 7], and also quantify relative transmission if it occurs. To date there is inconclusive evidence, although declines in transmission due to medical intervention over the last decades appear to rule out substantial animal transmission (see [37] for more discussion on this topic). A more detailed analysis of the presence and/or role of animal reservoirs is beyond the scope of the present study.

The model framework utilised in the present study is largely deterministic, yielding the same unobservable outputs every simulation for the same parameterisation – deterministic models can be considered to represent expected average dynamics. Whilst we do address some observational uncertainty through the overdispersion in the likelihood function and drawing model case outputs, the use of stochastic models which capture chance events in transmission and reporting will become increasingly important as infections approach zero. Other modelling studies utilising stochastic model formulations have found that, even at low reported case numbers and for relatively small populations (>2000 people), gHAT persists with high probability over long periods [5]. At the health zone level (∼100,000 people) model dynamics follow closely to deterministic ones until reaching extremely low case numbers [14]. We therefore consider that the deterministic model fits presented here would be similar to those obtained using a stochastic model variant in health zones with appreciable case reporting in the 2000s and regular good-coverage active screening. In health zones with limited case detection despite good screening coverage, a stochastic model would be more appropriate to obtain robust model fits. Future analysis should consider how the adaptive MCMC framework presented here could be built upon to fit stochastic model variants, especially if fits at smaller geographic scales (e.g. health areas ∼10,000 people) are required.

The automated adaptive MCMC approach taken will allow models to be fitted whenever necessary in a straightforward manner in response to the availability of new data or adaptations to the model.

This study is a necessary step towards providing modelling information which can assist in the formulation of policy appropriate to the varying needs across the country: the fits obtained here may now be used to simulate projections of the disease in to the

future under various different interventions. Once forecasts have been made, the results can be used to examine minimal strategies to achieve the 2030 elimination of transmission goal and also economic modelling can be used to ascertain cost-effective options, refining more general health economic analyses presented previously [38].

Conclusion

Supplementary Information

S1 Materials and Methods More detailed description of materials and methods.

S2 Supplementary Figures Additional results figures.

S3 Online Results Results for each health zone level fit can be viewed at `https://hatmepp.warwick.ac.uk/fitting/v1/`.

S4 Analysis Code Model code. The MATLAB code used to fit the model and perform the adaptive MCMC is available from `https://osf.io/ck3tr/?view_only=526344c12324492083db1e49c76136af`.

S5 Posterior samples Posterior parameter sets. The health zone-specific parameter sets obtained through this MCMC are available from `https://osf.io/ck3tr/?view_only=526344c12324492083db1e49c76136af`.

Acknowledgments

The authors thank PNLTHA for original data collection, WHO for data access (in the framework of the WHO HAT Atlas [1]), and Cyrus Sinai and Nicole Hoff from UCLA Fielding School of Public Health for providing health zone level shapefiles (current versions can be found at `https://data.humdata.org/dataset/drc-health-data`). This work was supported by the Bill and Melinda Gates Foundation (www.gatesfoundation.org) through the Human African Trypanosomiasis Modelling and Economic Predictions for Policy (HAT MEPP) project [OPP1177824] (C.H, R.E.C, P.B, K.S.R. and M.J.K.) and through the NTD Modelling Consortium [OPP1156227, OPP1186851 and OPP1184344] (K.S.R., E.K., S.E.F.S and M.J.K.). The funders had no role in study design, data collection and analysis, decision to publish, or preparation of the manuscript.

References

1. Franco JR, Cecchi G, Priotto G, Paone M, Diarra A, Grout L, et al. Monitoring the elimination of human African trypanosomiasis: Update to 2016. PLoS Neglected Tropical Diseases. 2018;12(12):e0006890.

2. World Health Organization. WHO interim guidelines for the treatment of gambiense human African trypanosomiasis; 2019.

3. Simarro PP, Cecchi G, Franco JR, Paone M, Diarra A, Ruiz-Postigo JA, et al. Mapping the capacities of fixed health facilities to cover people at risk of

gambiense human African trypanosomiasis. International Journal of Health Geographics. 2014;13(1):4.

4. Franco JR, Cecchi G, Priotto G, Paone M, Diarra A, Grout L, et al. Monitoring the elimination of human African trypanosomiasis at continental and country level: Update to 2018. PLOS Neglected Tropical Diseases. 2020;14(5):1–18.
5. Davis CN, Rock KS, Miaka EM, Keeling MJ. Village-scale persistence and elimination of gambiense human African trypanosomiasis. PLoS Neglected Tropical Diseases. 2020; p. e0007838.
6. Rock KS, Torr SJ, Lumbala C, Keeling MJ. Quantitative evaluation of the strategy to eliminate human African trypanosomiasis in the DRC. Parasites & Vectors. 2015;8(1):532.
7. Mahamat MH, Peka M, Rayaisse Jb, Rock KS, Toko MA, Darnas J, et al. Adding tsetse control to medical activities contributes to decreasing transmission of sleeping sickness in the Mandoul focus (Chad). PLoS Neglected Tropical Diseases. 2017;11(7):e0005792.
8. Simarro PP, Cecchi G, Jannin JG. The Atlas of human African trypanosomiasis: a contribution to global mapping of neglected tropical diseases. International Journal of Health Geographics. 2010;9:57.
9. Simarro PP, Cecchi G, Franco JR, Paone M, A D, Priotto G, et al. Monitoring the progress towards the elimination of gambiense human African trypanosomiasis. PLoS Neglected Tropical Diseases. 2015;9(6):e0003785.
10. Checchi F, Chappuis F, Karunakara U, Priotto G, Chandramohan D. Accuracy of Five Algorithms to Diagnose Gambiense Human African Trypanosomiasis. PLoS Neglected Tropical Diseases. 2011;5(7):e1233–15.
11. Rock KS, Torr SJ, Lumbala C, Keeling MJ. Predicting the impact of intervention strategies for sleeping sickness in two high-endemicity health zones of the Democratic Republic of Congo. PLoS Neglected Tropical Diseases. 2017;11:e0005162.
12. Rock KS, Pandey A, Ndeffo-Mbah ML, Atkins KE, Lumbala C, Galvani A, et al. Data-driven models to predict the elimination of sleeping sickness in former Equateur province of DRC. Epidemics. 2017;18:101–112.
13. Rock KS, Ndeffo-Mbah ML, Castaño S, Palmer C, Pandey A, Atkins KE, et al. Assessing Strategies Against Gambiense Sleeping Sickness Through Mathematical Modeling. Clinical Infectious Diseases. 2018;66(suppl. 4):S286–S292.
14. Castaño MS, Ndeffo-Mbah ML, Rock KS, Palmer C, Knock E, Miaka EM, et al. Assessing the impact of aggregating disease stage data in model predictions of human African trypanosomiasis transmission and control activities in Bandundu province (DRC). PLoS Neglected Tropical Diseases. 2020; p. e0007976.
15. OCHA Office for the Coordination of Humanitarian Affairs. Journees Nationales de Vaccination (JNV) Activities de vaccination supplementaire, RDC; Accessed May 2016. `https://data.humdata.org/about/license/legacy_hrinfo`.
16. The World Bank. Data:Democratic Republic of Congo; Accessed 2015. Available from: `https://data.worldbank.org/country/congo-dem-rep?view=chart`.

17. Rogers DJ. A general model for the African trypanosomiases. Parasitology. 1988;97:193–212.

18. Checchi F, Filipe JAN, Barrett MP, Chandramohan D. The natural progression of Gambiense sleeping sickness: What is the evidence? PLoS Neglected Tropical Diseases. 2008;2(12):e303.

19. Checchi F, Funk S, Chandramohan D, Haydon DT, Chappuis F. Updated estimate of the duration of the meningo-encephalitic stage in gambiense human African trypanosomiasis. BMC Research Notes. 2015;8(1):292.

20. Mpanya A, Hendrickx D, Vuna M, Kanyinda A, Lumbala C, Tshilombo V, et al. Should I Get Screened for Sleeping Sickness? A Qualitative Study in Kasai Province, Democratic Republic of Congo. PLoS Neglected Tropical Diseases. 2012;6(1):e1467.

21. Rock KS, Torr SJ, Lumbala C, Keeling MJ. Predicting the Impact of Intervention Strategies for Sleeping Sickness in Two High-Endemicity Health Zones of the Democratic Republic of Congo. PLOS NTD. 2017;.

22. Davis S, Aksoy S, Galvani AP. A global sensitivity analysis for African sleeping sickness. Parasitology. 2010;138(04):516–526.

23. Ravel S, Grebaut P, Cuisance D, Cuny G. Monitoring the developmental status of Trypanosoma brucei gambiense in the tsetse fly by means of PCR analysis of anal and saliva drops. Acta Tropica. 2003;88(2):161–165.

24. WHO. Control and surveillance of human African trypanosomiasis; 2013. 984.

25. Rock KS, Torr SJ, Lumbala C, Keeling MJ. Quantitative evaluation of the strategy to eliminate human African trypanosomiasis in the Democratic Republic of Congo. Parasites & Vectors. 2015;8(1):1–13.

26. Clausen PH, Adeyemi I, Bauer B, Breloeer M, Salchow F, Staak C. Host preferences of tsetse (Diptera: Glossinidae) based on bloodmeal identifications. Medical and Veterinary Entomology. 1998;12(2):169–180.

27. Lumbala C, Simarro PP, Cecchi G, Paone M, Franco JR, Mesu VKBK, et al. Human African trypanosomiasis in the Democratic Republic of the Congo: disease distribution and risk. International journal of health geographics. 2015;14(1):20.

28. FIND. Project Update: Kongo Central Driving elimination of human African trypanosomiasis in a challenging transboundary region; 2016. 984. Available from: https://www.finddx.org/wp-content/uploads/2016/03/HAT-KongoCentral-Eng-Jan2016.pdf.

29. Tirados I, Esterhuizen J, Kovacic V, Mangwiro TNC, Vale GA, Hastings I, et al. Tsetse Control and Gambian Sleeping Sickness; Implications for Control Strategy. PLoS Negl Trop Dis. 2015;9(8):e0003822. doi:10.1371/journal.pntd.0003822.

30. Tirados I, Stanton MC, Selby R, Mpembele F, Miaka EM, Boelaert M, et al. Impact of Tiny Targets on Glossina fuscipes quanzensis, the primary vector of Human African Trypanosomiasis in the Democratic Republic of the Congo. bioRxiv. 2020;doi:10.1101/2020.04.07.029587.

31. Haario H, Saksman E, Tamminen J, et al. An adaptive Metropolis algorithm. Bernoulli. 2001;7(2):223–242.

32. Gelman A, Roberts GO, Gilks WR. Efficient Metropolis jumping rules. Bayesian Statistics. 1996;5(599-608):42.

33. Gelman A, Rubin DB, et al. Inference from iterative simulation using multiple sequences. Statistical science. 1992;7(4):457–472.

34. Plummer M, Best N, Cowles K, Vines K. CODA: Convergence Diagnosis and Output Analysis for MCMC. R News. 2006;6(1):7–11.

35. Barrett MP, Burchmore RJ, Stich A, Lazzari JO, Frasch AC, Cazzulo JJ, et al. The trypanosomiases. The Lancet. 2003;362(9394):1469 – 1480. doi:https://doi.org/10.1016/S0140-6736(03)14694-6.

36. Büscher P, Cecchi G, Jamonneau V, Priotto G. Human African trypanosomiasis. The Lancet. 2017;390(10110):2397–2409.

37. NTD Modelling Consortium Discussion Group on Gambiense Human African Trypanosomiasis . Insights from quantitative and mathematical modelling on the proposed 2030 goal for gambiense human African trypanosomiasis (gHAT) [version 2; peer review: 1 approved, 1 approved with reservations]. Gates Open Res. 2020;3(1553).

38. Sutherland CS, Stone CM, Steinmann P, Tanner M, Tediosi F. Seeing beyond 2020: an economic evaluation of contemporary and emerging strategies for elimination of Trypanosoma brucei gambiense. The Lancet Global Health. 2017;5(1):e69–e79.

Click here to access/download
Supporting Information
HAT_MEPP_fitting_Supp_Info.pdf
