# Supplementary material for: Quantifying epidemiological drivers of gambiense human African Trypanosomiasis across the Democratic Republic of Congo
Source: PLoS Comput Biol. 2021 Jan 29;17(1):e1008532. doi: 10.1371/journal.pcbi.1008532 (PMC7899378; doi:10.1371/journal.pcbi.1008532)
Supplement: Attachment — Submitted filename: response.pdf [file pcbi.1008532.s008.pdf]

# PCOMPBIOL-D-20-01092: Response to reviewers

We were very pleased to receive the Minor Revisions editorial decision and the helpful and supportive comments and suggestions made by the three reviewers.

All three reviewers intimated that, in one area or another, more should be said about policy decisions which might result from these analyses. This piece of work primarily outlines a framework for the analysis of historical data, focusing on estimating parameters associated with the model and discussing what this means in terms of success of past interventions. We have now included policy recommendations around data collection and digitisation. Our parameter estimates can be used to make future predictions under various gHAT control/elimination strategies, which can subsequently be included in economic analysis. It is these future predictions and economic evaluations, which do not form part of this paper, which will have implications for specific gHAT intervention strategies. We do not wish to pre-empt the results of other papers from the group (eg, https://doi.org/10.1101/2020.07.03.20145847 and https://doi.org/10.1101/2020.08.25.20181982) which build upon the foundations set out in the current manuscript, with each representing a substantial piece of research in its own right.

We have attempted to make the structure of our research programme, and the vital role within it of this work, clearer in the Introduction and Discussion:

> **Added at the end of the Introduction:**
> *There are three major outputs from this study. Firstly, the parameterisation of our gHAT transmission model which allows future predictions to be made considering different gHAT intervention strategies [8]. With future predictions based on realistic, localised epidemiological parameters; economic evaluations of the cost-effectiveness of different approaches to the control of gHAT are possible [9]. Secondly, the evaluation of the effectiveness of interventions against gHAT in DRC across the period 2000 to 2016. Lastly, but potentially of most value to our research going forwards, the provision of a rapid, repeatable model fitting framework to facilitate future research around the model presented here and other variants of it.*

We also note that in revising our manuscript we have also made minor improvements to the way in which missing data were handled (affecting 4 health zones), and how we dealt with small effective sample sizes. The relevant text and all maps/values in the analysis have been updated to reflect this, as has the associated GUI, for which there is now a version 2 (https://hatmepp.warwick.ac.uk/fitting/v2/).

**Reviewer 1:**

I have several, somewhat related, comments that the authors may like to consider:

1. It is not clear to me what authors think about the role tsetse flies have in this whole story. While the tsetse part of the mathematical model is extensive, and carefully developed, we are then told (Table 1, bottom) that the “Value of BV [the tsetse birth rate] is chosen to maintain constant population size without interventions.”

   By “interventions” I understand that the authors mean *purposeful* interventions, specifically aimed at killing tsetse. In the present case, this was limited to the use of tiny targets in a single health zone, Yasa Bonga, where it was assumed that tsetse control operations led to a 90% reduction in fly numbers.

   But then that is the last we here of the beloved fly. And we are left to wonder whether, or not, tsetse are indeed drivers of g-HAT in the DRC? There is no mention of this question in the Discussion.

   We have added two additional paragraphs to the discussion about the role of tsetse.
   “In this study we have been able to estimate the relative vector-to-host ratio for different regions through our adaptive MCMC. Maps of $m_{eff}$ are shown in SI “S2 Posteriors of fitted parameters”, Fig S2.8, however these alone do not tell the whole story and are not necessarily reflective of where one might find the highest density of flies in DRC. In particular the high-/low-risk structure of the model (associated with parameters $k_1$, the proportion of the population who are low risk, and $r$ the relative exposure of high-risk individuals) is intimately entwined with the relative vector-to-host ratio. It would, therefore, be possible in this framework to have a comparatively low $m_{eff}$ in a health zone, but high $r$ due to dense pockets of tsetse habitat which only high-risk people are exposed to; overall this could lead to a high burden health zone due to population-level heterogeneity in risk.

   Despite the complex nature of inferring the role of tsetse, we do examine a single case where deliberate vector control has been deployed during the time period of our dataset. In Yasa Bonga, tiny targets were used from 2015 and although it is not strongly apparent by simply looking at case detections in 2016, we estimate that transmission in the region has sharply fallen from around 22.6 (95% CI: 11.2--41.2) new infections per year in 2015 to 1.3 (95% CI: 0.6--2.5) in 2016. Due to the slow progression of the disease, it would be expected to take several years of case data until there is a marked decline in both active and passive case detections. In future studies, more years of human case and tsetse monitoring data from Yasa Bonga and other health zones, which have subsequently begun tsetse control, will help to provide deeper insight into the role of these vectors in transmission dynamics.”

2. And if, in fact no tsetse control operations were carried out in an area, is it even necessary to add the complexity of tsetse biology into the model? Since BV [the

tsetse birth rate] is then manipulated to keep tsetse populations constant would it not be simpler to have trypanosomiasis “rain down” on humans at a fixed rate? This would remove from the model a large number of parameters that are unnecessary, except when tsetse control is *actively* being pursued.

Vector control (VC), via deployments of tiny targets, forms one of the main strategies under consideration in our modelling work. However, there was little historical case data associated with VC, with VC having been implemented in only one health zone in DRC before the end of the period for which data were available to us. In order that VC can be considered in future predictions under this and other models, it is necessary to include the vector population dynamics.

3. On the other side of the coin, tsetse distribution has been markedly influenced by matters entirely unrelated to formal tsetse control operations. Indeed, it is the view of David Rogers and his co-workers that “autonomous” tsetse control has perhaps been at least, perhaps even more, effective than the purposive variety. Is it not entirely possible that the combined effects of the clearing of forest and killing of wild animals has negatively impacted tsetse distribution, and thereby reduced the incidence of g-HAT?

You raise an interesting point that unintentional suppression of tsetse populations has likely been occurring in various settings. As we have no data on tsetse population trends in DRC, nor other data to suggest where this may have occurred, we have not included it in our analysis. Given the model fits we have achieved and recent monitoring due to efforts to intentionally control tsetse in parts of Bandundu province, we believe that this was a justifiable assumption to make. Clearly this will not be true across the continent and deforestation and urbanisation some locations will have had substantial impact. We specially excluded many health zones from Kinshasa province due to the uncertain nature of where transmission occurred that was reported in these regions and the urban setting.

We have added the following paragraph in the discussion:
“The gHAT model presented is an explicitly Ross-Macdonald-style, host-vector model capable of simulating the impact of vector population size changes on transmission to humans. This was introduced to enable the simulation of intentional vector control which was deployed in Yasa Bonga health zone from 2015 and so that future modelling using this framework could explore the possible impact of vector control in other locations. We did not, however, consider other sources of fluctuation of tsetse populations, although it is noted that anthropogenic change in particular could result in loss of tsetse habitat and inadvertently reduce tsetse populations. There are limited data available on temporal changes in tsetse density across DRC to inform such an analysis, however it could be a potential avenue for future research in areas of known deforestation or urbanisation.”

4. Another factor in such discussions concerns the view of Welburn, Molyneux and Maudlin (2016) that, really, g-HAT has nothing to do with tsetse. This view may be regarded by many as quaint, bordering on the quixotic. Nonetheless, the authors [particularly the first and last] are leading luminaries in the world of trypanosomes and trypanosomiasis – and the authors might feel that it is a little high-handed to ignore their views entirely.

   I am not suggesting that, in responding to the above points, the authors need to change the model or the body of Results section. But the matters raised might, nonetheless, merit some space in the Discussion….

   We have added the follow paragraph into the discussion:
   "One published review [40] has previously suggested that the primary transmission mechanism of gHAT is not tsetse. In the present modelling study, for the most part, it would be unlikely to make any difference to our results to assume human-to-human transmission as we assume stable vector populations which could be readily substitute by a quasi-equilibrium assumption or even modelled as a contagion without substantial quantitative differences. However, where vector control is implemented, our assumption that gHAT is a vector-borne infection would produce very different results compared to assuming no or limited tsetse transmission. This would be very pronounced if the model is subsequently used to make projections of future vector control impact. We are reassured by various other recent studies that our tsetse-transmission assumption is valid as (i) the host-vector gHAT model predictions for regions with vector control appear to match the case reductions well [7] and (ii) the natural experiment which occurred in Guinea during the 2014--15 West African Ebola outbreak found that even following interruption of medical screening activities, regions that has vector control remaining in situ had much lower case burden following resumption of screening than those without [41, 42]."

5. Finally, and most importantly, I do feel that the authors need to take a stand on how g-HAT should be handled in the DRC. Do they, or do they not, think that vector control is necessary, desirable, cost-effective etc. If not, do they feel – for example – that the problem will fall of its own weight, so that then is passive case finding is sufficient? Or do they feel that a mixture of passive and active case detection is called for? Some of these matters may have been touched on explicitly and others implicitly, but I feel that a more substantive statement is called for to wrap up the paper.

   We think that giving a true answer to this question is beyond the scope of this paper, given its focus on fitting methods and assessment of historic trends, however we have attempted to hint at this question and follow on work which precisely addresses this (see MedRxiv for the follow on work on projections and health economics:
   https://www.medrxiv.org/content/10.1101/2020.07.03.20145847v1 and

https://www.medrxiv.org/content/10.1101/2020.08.25.20181982v1). In the conclusion we have added;

"This country-wide analyses has given an insight into why transmission may persist in some locations more than others, for example the results indicate that passive detection rates were higher in Equateur province in the early 2000s than in Bandundu province and teamed with active screening this resulted in a more marked decline in both cases and transmission. As well as interventions, epidemiological factors, including human behaviour, are likely driving infection differences: the relative risk of high-risk people compared to low-risk appears to be greater in known persistent regions of Bandundu -- including the health zones of Kwamouth, Bolobo, Mushie and Bagata -- compared to other provinces.

This finding raises the question of whether current medical-only interventions are sufficient to reduce transmission to the point of interruption before 2030 in all regions, or whether other approaches may be needed in locations with moderate but less substantial reductions. Indeed, previous modelling for specific health zones in DRC has suggested intensified interventions such as targeting high-risk groups in active screening or vector control may be needed to speed up progress and meet the 2030 goal [13, 15]. A larger challenge is to identify where this may be needed across the country, and where should be prioritised for bolstered interventions."

**Minor matters**

I have returned a copy of the MS, highlighting various bits of text where the presentation could be improved by the judicious addition of commas, definite, or indefinite articles, or just attention to matters of clumsiness, general ugliness or lack of clarity of meaning. I don't have strong feelings about any particular case: up to the authors. But a general tidy up is, I feel, in order for what is such a high-quality paper.

Thank you for the highlighted PDF, we greatly appreciate the effort made and attention to detail applied. We believe we have addressed the highlighted areas, however they are too numerous to list here and refer you to our marked-up PDF in which changes are indicated.

**References**

**Bourne, D., Reid, R.S., Rogers, D.J., Snow, W.F. & Wint., W.** (2001) *Environmental change and the autonomous control of tsetse and trypanosomiasis in sub-Saharan Africa.* Oxford, Environmental Research Group Oxford Limited. 248 + xi *pp*.

**Welburn, S.C., Molyneux, D.H., Maudlin, I.** (2016) Beyond tsetse – implications for research and control of human African trypanosomiasis epidemics. *Trends in Parasitology* **32**, 230-241.

Both of these references are now cited as part of the changes described above

**Reviewer 2:**

Major points:

1. It would be helpful to provide more detail regarding the manual tuning of the overdispersion parameters (lines 219-221). Was there a certain criterion used to deem the fit appropriate?

Paragraph modified to read:

Consequently larger ρ yields more overdispersion. To avoid overfitting, the overdispersion parameters were left fixed at a value appropriate for a health zone level fit across MCMC runs (Table 1). The value of ρ was chosen based on the median of the log posterior probability distribution achieved from MCMC runs with ρ fixed at a range of values for two example health zones.

2. Was the health zone level data that was used for model fitting independent from the province level data described on lines 226-227? The data described in the Data section doesn't refer to province level data, so I am wondering if this is a separate dataset used for validation purposes, or if the health zone and province level data come from the same raw dataset and this step was more of an internal check for consistency.

Internal check for consistency. Particularly important where there was passive surveillance/detection improvement modelled, with the change occurring at different times in different health zones, i.e. in former Bandundu province.

3. Similarly, there is reference to model fitting to province-level data on lines 234-235. Is this a distinct dataset? If not, what is the rationale for fitting at both the province and health zone levels?

The data available to us (HAT Atlas, 2000-2016) do not contain staging information for the vast majority of health zones until 2015 and 2016. However, the paper of Lumbala et al (2015) includes province-level data from 2000-2012. We augmented these data with aggregated HAT Atlas data for 2013-2016 and fitted the model to them in order that these data could inform the health zone-level priors.

We have changed the paragraph around lines 234-5 to read:

> *Informative priors were used for most parameters. There was little information on the stage of the disease in the data, essentially none before 2015. Former-province level staged case numbers were available from 2000-2012 [28] and we augmented these with our data aggregated to the former-province level for the years 2013 to 2016. The model was fitted to these staged former-province level data and informative priors for the parameters relating to treatment rates from stages 1 and 2 ($\eta_H^{post}$ and $\gamma_H^{post}$), and*

*improvement in passive detection ($\eta_{Hamp}$, $\gamma_{Hamp}$ and $d_{steep}$) were based on the respective posterior distributions from these analyses.*

4. I am curious about the rationale for choosing a single random value from the posterior distribution of R0 for showing geographical variation (line 318-322, Figure 6). Would a standardized sample across all locations, such as taking the median of the posteriors, be more representative?

There is no rationale used for choosing a single random value to represent geographical variation, as we do not use a single value to represent each geographical unit. The geographical unit of interest here is the health zone and the locations of the hexagons within the health zone are meaningless. Figure 6 has the health zones partitioned into small hexagons, or partial hexagons at health zone boundaries, filled with random samples from the health zone-level R0 posterior distribution. So, rather than a health zone being filled with a single value (eg, the median) it is filled in a way that reflects the variability of the estimates of R0 within that health zone. The colour variation reflects the variation in R0, the most frequent colour reflects the mode.

5. The discussion surrounding model limitations and future applications is really nice, but it would be great for more to be included about the outcomes of the modeling done in the paper. The takeaway message and major findings could be stronger in this section. There were some interesting analyses done in this work that were presented well in the Results section and could be better highlighted in the Discussion.

We have now included a separate conclusions section to focus on takeaway messages in addition to the discussion. We highlight, in particular, the role of passive surveillance and population behaviour/risk as key drivers.

Minor points:

1. Overall, the data is explained clearly, and the model structure and calibration procedure are well described. As it is currently described in lines 126 and 127, it was not immediately clear to me that the population was divided into low- and high-risk human categories. I interpreted this as low- and high-risk scenarios until I looked at Table 2 more closely. It might be helpful to draw attention to the parameters k1 and k4 in the text.

Thank you for drawing attention to this. As the work has been presented before we did not carefully consider the presentation of the model from the perspective of a reader coming across it for the first time. We have now added in more details regarding the high-/low-risk categories and subscripts as well as tidying up other parts of the model description.

2. Since non-reservoir animals are not explicitly modeled, the non-reservoir animal compartment in Fig 3 could be removed for clarity.

We understand this viewpoint, however as the parameter f_H (proportion of bites on humans) is necessarily related to other sources of blood meals (1-f_H) we wanted to be clear that we do factor this into our calculation. This is consistent with our previously

published model diagrams, and is pertinent when we consider model variants with reservoir animals.

3. Was there intended to be a conclusion section? There is currently a header with no content.

We have now used some paragraphs that were in the discussion along with some new text to form a conclusions section.

4. The paper could benefit from proofreading for phrasing and grammar. There are a few run-on and awkwardly phrased sentences, but overall, the paper is nicely written and organized.

We hope that some improvements have been made in this area.

**Reviewer 3:**

Although the rationale of the study is clearly explained very well, the discussion section mainly focussed on the methodological aspects of the work, which left me wondering "so what does it all mean?". Answering this question would make the paper more interesting for a wider public, both within the field of HAT control as well as other infectious diseases.

Methods section, paragraph "gHAT infection model": it would be helpful to start this paragraph by summarising some general characteristics of the model, as readers may not be familiar with earlier work based on this model. E.g., I presume that the model is deterministic and somehow stratified (given the index $i$ in equation (1), but stratified by what? Age? Risk group?).

We have added some additional clarification here that the subscripts denote low-risk participating (i=1) and high-risk non-participating (i=4) classes. We do not use i=2 or i=3 here, although for consistency with prior (and future!) publications we retain the notation. We agree that for readers unfamiliar with the previously published model variants, this was not fully explained.

After reading Table 1, Figure 3, and equation 1, I understand that the model represents a closed population of stable size (might be good to mention that somewhere) and that the only human mortality considered is "natural mortality" based on an average expected lifespan of 50 years with exponentially distributed lifespans, correct? Why does the model not include disease-specific mortality? It seems an important element of the process of transmission and how that is reflected in actual reported case numbers. People who are not detected, presumably die eventually, correct? This is now implicitly captured by the process that people who leave the infected/infectious compartment (regardless of cure or mortality) go to the recovered compartment, which has an average duration of ~167 days.

We now mention the closed population of humans, and discuss how disease-induced mortality is incorporated in the "stage 2 exit rate" parameter, gamma_H. Thank you for highlighting this as it was not very clear from the table description.

Equation (1) includes a symbol f_T(t), for which I'm not sure what it means. It is not mentioned in either Table 1 or 2.

The following paragraph has been added:
Vector control is included in Eq (1) as $f_T(t)$, the probability of a fly both hitting a tiny target and subsequently dying at time $t$. The value of $f_T(t)$ is dependent on the population reduction achieved by any vector control performed. For Yasa Bonga health zone, the only health zone in which vector control took place prior to the end of the data collection period, a 90\% reduction in tsetse population in the first year after biannual deployment of tiny targets was introduced [27]; more details are given in the SI (S1 Materials and Methods) about the functional form of $f_T(t)$, which was originally presented elsewhere [13].

The use of only two MCMC chains and application of the convergence criterion R_between < 1.5 to only the adaptive phase seems risky. I would strongly recommend to run four chains (not just two) and to also check that R_between < 1.1 and ideally 1.05 for the final sample of posterior samples (i.e. not just the adaptive phase). Also, maybe the "1.5" is a typo in the manuscript, because Fig 4 shows R_between <1.05 for all districts at the end of the adaptive phase.

We agree that the use of R_between < 1.5 as the criteria to begin sampling would be a very risky approach, if this was the sole criterion. However, the requirement for R_within to be < 1.1 for all parameters mitigates this risk.The reviewer is correct in pointing out that we could have tighten the restriction on R_between without any change in results

We are confident that 2 chains are sufficient for our purposes here. Although we have observed analyses under other models (ie the Warwick HAT model, but more highly parameterised variants than the model 4 variant used here) in which the chains did not converge to the same target distribution, the continuous nature of our fitted parameters has, to date, made this relatively straightforward to address when necessary. Although two chains are reported in the manuscript, many similar chains with minor variations were run in the draft stages and no multimodality was observed.

Plots of parameter value against sample number are provided below for Kwamouth and Tandala health zones. Note that Kwamouth was one of the health zones that initially had ESS below 1,000 and has had some additional sampling and thinning and is also in Bandundu, and so has more parameters being estimated than, eg, Tandala. It might be expected therefore that Kwamouth would be an area where any issues should be visible.

Figure 4 does report R_between for the final set of posterior samples. The caption for this figure and the sentence referring to it have been updated to reflect this.

I would like to challenge the authors to revise the discussion into a more coherent unit and perhaps make some recommendations. Only lines 358-366 comment a little on what was actually found in terms of results, but left me wondering "so what does it actually mean?"

We have added a few recommendations as suggested including on (i) reporting of stage case data to inform on the performance of the passive surveillance system, (ii) reporting of numbers of passive test performed, (iii) age and gender profiles of cases and screened populations, and (iv) the value of data around vector control, from both entomological and human-exposure surveys.

“We note that the health-zone-level longitudinal case data typically did not have staging information associated with them prior to 2015. The staging information for 2015 and 2016 was highly informative in quantifying passive detection improvements when combined with provincial-level staged data published separately [28]. We reiterate the recommendation of Castaño {\it et al.} [16], in collecting and digitising staged case data whenever possible to enable continued assessment of the passive surveillance system.”

We also allude to future work which should address how some health zones with less success in driving down transmission could modify strategy in the future in our new conclusion section:
“These country-wide analyses have given an insight into why transmission may persist in some locations more than others, for example the results indicate that passive detection rates were higher in Equateur province in the early 2000s than in Bandundu province and teamed with active screening this resulted in a more marked decline in both cases and transmission. As well as interventions, epidemiological factors, including human behaviour, are likely driving infection differences: the relative risk of high-risk people compared to low-risk appears to be greater in known persistent regions of Bandundu -- including the health zones of Kwamouth, Bolobo, Mushie and Bagata -- compared to other provinces.

This finding raises the question of whether current medical-only interventions are sufficient to reduce transmission to the point of interruption before 2030 in all regions, or whether other approaches may be needed in locations with moderate but less substantial reductions. Indeed, previous modelling for specific health zones in DRC has suggested intensified interventions such as targeting high-risk groups in active screening or vector control may be needed to speed up progress and meet the 2030 goal [13, 15]. A larger challenge is to identify where this may be needed across the country, and where should be prioritised for bolstered interventions.”

The conclusion section at the end of the discussion is empty!

We have now restructured and added a separate conclusions section.

Minor comments:

- Throughout the manuscript, some paragraphs are extremely short, sometimes even only one sentence. Consider to integrate into adjacent paragraphs.
- Author summary: consider rephrasing last sentence which has “future” in it twice.

Changed to:
The model fitting presented here will allow predictions of gHAT under alternative intervention strategies to be performed in future studies.

- Introduction, fourth paragraph: consider reversing the order in which the treatments are explained (i.e. set them in chronological order) such that the “(still do today)” is not needed.

Simplified that sentence and removed “(still do today)”.

- Methods, data section:
    - please clarify whether the HAT Atlas case data includes information about how cases were detected (passive vs. active screening) and whether that information was used when aggregating case numbers to the health zone level.

Added references to surveillance type (passive or active screening) when describing the WHO data in paragraph 1 of this section, and that aggregation was by health zone, year and surveillance type in paragraph 3.

- Figure 1 suggests that there is no transmission in Kinshasa, whereas Figure 2 suggests that after all cases were reported there. It would be good to be explicit about whether or not inference was done for Kinshasa.

The following addition was made to the final paragraph of the Methods, Data section:

Some cases were reported in urban Kinshasa (Fig 2), however these are believed to be the result of infections that occurred elsewhere and there is assumed to be no transmission within urban Kinshasa (Fig 1) and hence no model fitting was performed for those health zones.

- Table 2: it took me a while to under what was meant by “Relative treatment rate from stage 2 factor, pre-1998”, which I only got after reading the next line “Treatment rate from stage 2, pre-1998” and the associated definition in the “percentiles” column (which is not a very logical place for this information). Suggestion: switch the two rows and put the definition of “Treatment rate from stage 2, pre-1998” in the “prior” column. Also, consider removing the symbol “X” and tilde sign which are used in all rows of the column “prior”; I got confused for a moment by the use the same symbol for all parameters.

Removed the ‘X ~’ from all prior distribution definitions. Also removed the lines from the table relating to k_4 and and the treatment rate from stage 2 pre-1998, as these are not directly fitted parameters in their own right. As a result, added the following sentences to the first paragraph discussing this table:

Some parameters of the model are not fitted themselves but are functions of other parameters. The proportion of high-risk people in the population was calculated as $k_4 = 1-k_1$. The treatment rate from stage 2 pre-1998 was assumed to be less than that

achieved post-1998, and was therefore calculated as $\gamma_{H}^{\rm pre}=b_{\gamma_{H}^{\rm pre}}\gamma_{H}^{\rm post}$, where $\gamma_{H}^{\rm post}$ was the treatment rate from stage 2 post-1998 and $b_{\gamma_{H}^{\rm pre}}$ is a fitted value in the range zero to one.

- Lines 144-147: parameters s_1 and s_4 are not mentioned in either Table 1 or 2.

s_1 and s_4 represent the availability/attractiveness of hosts to tsetse. The parameter r is fitted and s_4 takes this value, high-risk humans being r times more likely to be bitten than low-risk humans. Changed the sentence referring to s_1 and s_4 to:
If $s_i$ is the relative availability/attractiveness of different host types and high-risk humans are assumed to be $r$-fold more likely to receive bites, then $s_1=1$ and $s_4=r$

- Lines 195-196: rephrase "…which is especially important as there become very few cases remaining."

Changed to "...which is especially important as elimination is approached and very few cases remain."

- Log-likelihood function: index *i* is used here to indicate time (year) whereas in the equations it's used to indicate risk group; I suggest to use index *t* for time to avoid confusion.

Change made.

- Lines 222-225: I don't understand the need for a negative binomial likelihood; why not use the regular likelihood as for the rest of the data points and simply drop the terms associated with screening if that did not happen?

We have now changed the way in which we estimate missing screening numbers in the four health zones in which that occurs. A hierarchical model is assumed for the missing values, the probability of a positive test result being calculated from the current sample estimates. The prior and proposal distributions (negative binomial) for the missing screening number values are the same and cancel from the Likelihood. Lines 222-225 have been re-written accordingly.

- Line 256: I hope that during the sampling phase, the proposals were no longer drawn adaptively! The text suggests that adaptive proposals were still being done.

Adaptation does continue during the sampling phase, however the modifications to the covariance matrix decrease in size as the number of samples increases. Roberts and Rosenthal (Journal of Applied Probability, 2007, 44:458–475) consider two requirements for an adaptive algorithm to converge to the correct target distribution: diminishing adaptation and a technical condition called "containment". Our algorithm

satisfies diminishing adaptation because the amount that the proposal covariance matrix can change at each iteration shrinks to zero. The reviewer is correct in pointing out that the simplest way to satisfy the diminishing adaptation condition is to stop the adaptation after the burn-in, but this is not the only way to satisfy the condition. The technical condition of containment is satisfied as long as the proposal scaling parameter $\lambda$ converges to a positive constant (which occured in all of our chains). The observed convergence of the analyses implies that containment has been met in practice.

On the following pages we include plots of parameter value against posterior sample number, for each chain, for the example health zones used in the manuscript (Kwamouth and Tandala).

There remains some evidence of autocorrelation, which is unsurprising given that (a) the requirement was to have an effective sample size of 1,000 across the 2 chains of 1,000 samples, and (b) there are a lot of parameters interacting.

There is no obvious indication either that the chains are not in the same area of parameter space (see your earlier comment on the levels of the convergence criteria) or that there is noticeable change in target distribution across the sampling period (as might be expected if adaptation had not diminished).

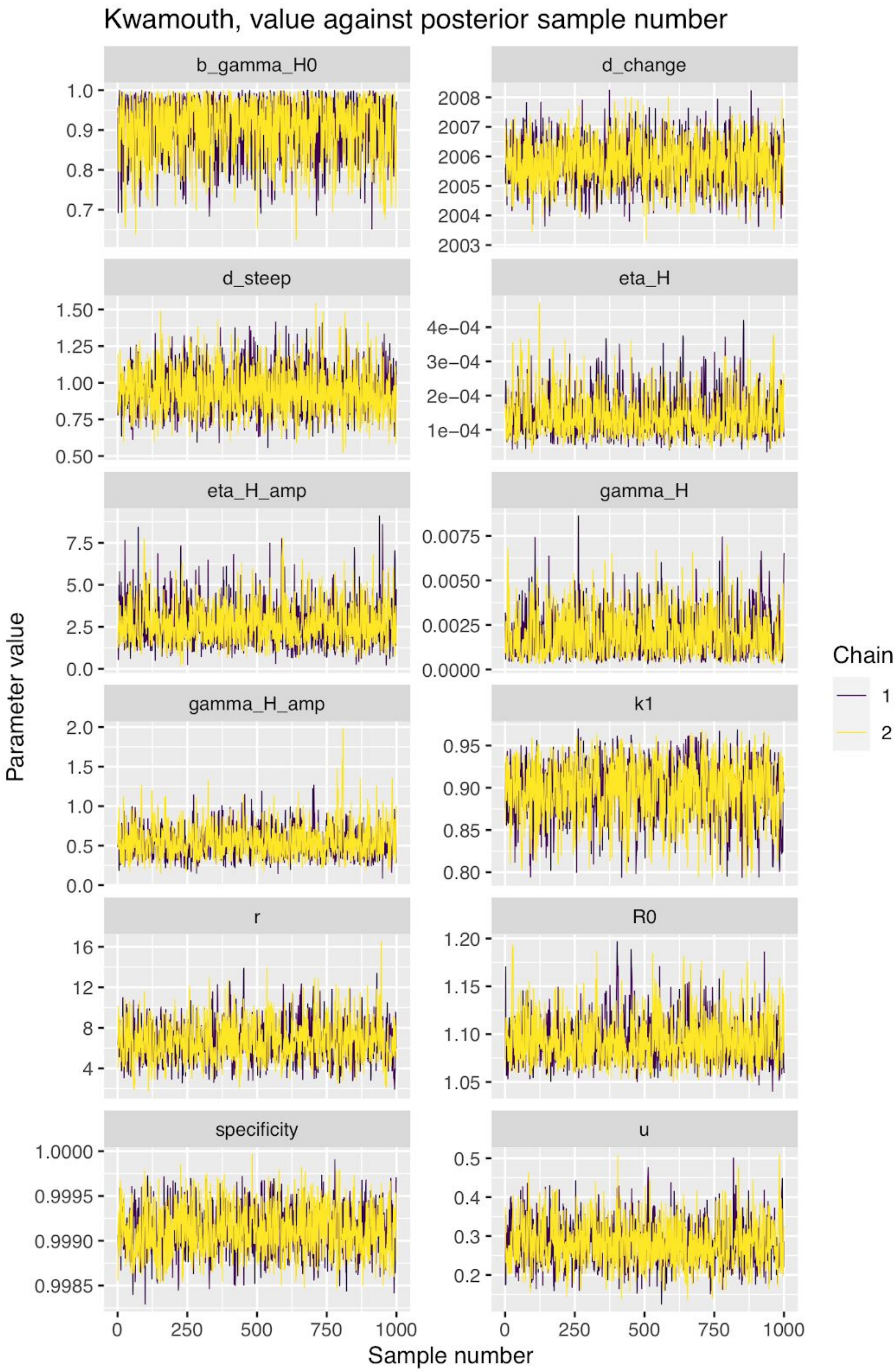

Kwamouth, value against posterior sample number
b_gamma_H0
d_change
d_steep
eta_H
eta_H_amp
gamma_H
gamma_H_amp
k1
r
R0
specificity
u
Parameter value
Sample number
Chain
1
2

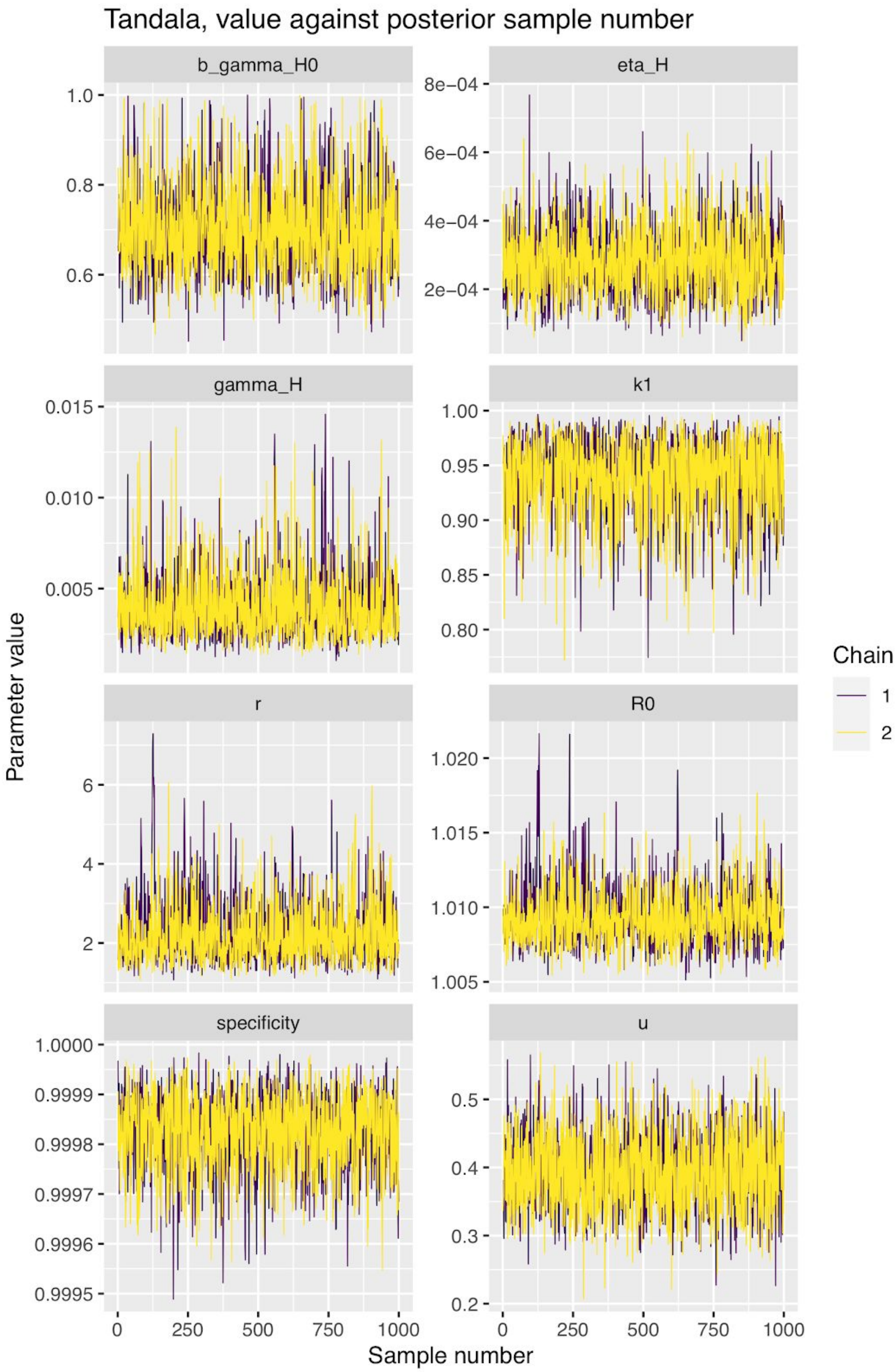
Tandala, value against posterior sample number
b_gamma_H0
eta_H
gamma_H
k1
r
R0
specificity
u
Parameter value
Sample number
Chain
1
2
