# Supplementary material for: Quantifying epidemiological drivers of gambiense human African Trypanosomiasis across the Democratic Republic of Congo
Source: PLoS Comput Biol. 2021 Jan 29;17(1):e1008532. doi: 10.1371/journal.pcbi.1008532 (PMC7899378; doi:10.1371/journal.pcbi.1008532)
Supplement: Attachment — Submitted filename: Crump et al Rock 2020 gHAT in DRC Review of paper Hargrove 22 Jul 2020.docx [file pcbi.1008532.s005.docx]

**Review of the paper:**

**Quantifying epidemiological drivers of gambiense human African Trypanosomiasis across the Democratic Republic of Congo**

**Ronald E Crump, Ching-I Huang, Ed Knock, Simon E F Spencer, Paul**

**Brown, Erick Mwamba Miaka, Shampa Chancy, Matt J Keeling, Kat S Rock**

This is a thorough, impressive and highly professional piece of work and should clearly be published. The statistical analysis and mathematical modelling impressive and it is difficult to find fault with them.

I have several, somewhat related, comments that the authors may like to consider:

1. It is not clear to me what authors think about the role tsetse flies have in this whole story. While the tsetse part of the mathematical model is extensive, and carefully developed, we are then told (Table 1, bottom) that the “Value of BV [the tsetse birth rate] is chosen to maintain constant population size without interventions.”
   By “interventions” I understand that the authors mean *purposeful* interventions, specifically aimed at killing tsetse. In the present case, this was limited to the use of tiny targets in in a single health zone, Yasa Bonga, where it was assumed that tsetse control operations led to a 90% reduction in fly numbers.

   But then that is the last we here of the beloved fly. And we are left to wonder whether, or not, tsetse are indeed drivers of g-HAT in the DRC? There is no mention of this question in the Discussion.
2. And if, in fact no tsetse control operations were carried out in an area, is it even necessary to add the complexity of tsetse biology into the model? Since BV [the tsetse birth rate] is then manipulated to keep tsetse populations constant would it not be simpler to have trypanosomiasis “rain down” on humans at a fixed rate? This would remove from the model a large number of parameters that are unnecessary, except when tsetse control is *actively* being pursued.
3. On the other side of the coin, tsetse distribution has been markedly influenced by matters entirely unrelated to formal tsetse control operations. Indeed, it is the view of David Rogers and his co-workers that “autonomous” tsetse control has perhaps been at least, perhaps even more, effective than the purposive variety. Is it not entirely possible that the combined effects of the clearing of forest and killing of wild animals has negatively impacted tsetse distribution, and thereby reduced the incidence of g-HAT?
4. Another factor in such discussions concerns the view of Welburn, Molyneux and Maudlin (2016) that, really, g-HAT has nothing to do with tsetse. This view may be regarded by many as quaint, bordering on the quixotic. Nonetheless, the authors [particularly the first and last] are leading luminaries in the world of trypanosomes and trypanosomiasis – and the authors might feel that it is a little high-handed to ignore their views entirely.

   I am not suggesting that, in responding to the above points, the authors need to change the model or the body of Results section. But the matters raised might, nonetheless, merit some space in the Discussion….
5. Finally, and most importantly, I do feel that the authors need to take a stand on how g-HAT should be handled in the DRC. Do they, or do they not, think that vector control is necessary, desirable, cost-effective etc. If not, do they feel – for example – that the problem will fall of its own weight, so that then is passive case finding is sufficient? Or do they feel that a mixture of passive and active case detection is called for? Some of these matters may have been touched on explicitly and others implicitly, but I feel that a more substantive statement is called for to wrap up the paper.

**Minor matters**

I have returned a copy of the MS, highlighting various bits of text where the presentation could be improved by the judicious addition of commas, definite, or indefinite articles, or just attention to matters of clumsiness, general ugliness or lack of clarity of meaning. I don’t have strong feelings about any particular case: up to the authors. But a general tidy up is, I feel, in order for what is such a high-quality paper.

**References**

**Bourne, D., Reid, R.S., Rogers, D.J., Snow, W.F. & Wint., W.** (2001) *Environmental change and the autonomous control of tsetse and trypanosomiasis in sub-Saharan Africa.* Oxford, Environmental Research Group Oxford Limited. 248 + xi *pp*.

**Welburn, S.C., Molyneux, D.H., Maudlin, I.** (2016) Beyond tsetse – implications for research and control of human African trypanosomiasis epidemics. *Trends in Parasitology* **32**, 230-241.

John Hargrove

Somerset West

22 July 2020
