# Supplementary material for: Quantifying epidemiological drivers of gambiense human African Trypanosomiasis across the Democratic Republic of Congo
Source: PLoS Comput Biol. 2021 Jan 29;17(1):e1008532. doi: 10.1371/journal.pcbi.1008532 (PMC7899378; doi:10.1371/journal.pcbi.1008532)
Supplement: Attachment — Submitted filename: PCOMPBIOL-D-20-01092_comments LC.docx [file pcbi.1008532.s007.docx]

In general, the manuscript is well written and provides a pleasant read, although it could benefit from some extra clarification of methodological matters in the methods section (see details below). Although the rationale of the study is clearly explained very well, the discussion section mainly focussed on the methodological aspects of the work, which left me wondering “so what does it all mean?”. Answering this question would make the paper more interesting for a wider public, both within the field of HAT control as well as other infectious diseases.

Methods section, paragraph “gHAT infection model”: it would be helpful to start this paragraph by summarising some general characteristics of the model, as readers may not be familiar with earlier work based on this model. E.g., I presume that the model is deterministic and somehow stratified (given the index *i* in equation (1), but stratified by what? Age? Risk group?).

After reading Table 1, Figure 3, and equation 1, I understand that the model represents a closed population of stable size (might be good to mention that somewhere) and that the only human mortality considered is “natural mortality” based on an average expected lifespan of 50 years with exponentially distributed lifespans, correct? Why does the model not include disease-specific mortality? It seems an important element of the process of transmission and how that is reflected in actual reported case numbers. People who are not detected, presumably die eventually, correct? This is now implicitly captured by the process that people who leave the infected/infectious compartment (regardless of cure or mortality) go to the recovered compartment, which has an average duration of ~167 days.

Equation (1) includes a symbol f_T(t), for which I’m not sure what it means. It is not mentioned in either Table 1 or 2.

The use of only two MCMC chains and application of the convergence criterion R_between < 1.5 to only the adaptive phase seems risky. I would strongly recommend to run four chains (not just two) and to also check that R_between < 1.1 and ideally 1.05 for the final sample of posterior samples (i.e. not just the adaptive phase). Also, maybe the “1.5” is a typo in the manuscript, because Fig 4 shows R_between <1.05 for all districts at the end of the adaptive phase.

I would like to challenge the authors to revise the discussion into a more coherent unit and perhaps make some recommendations. Only lines 358-366 comment a little on what was actually found in terms of results, but left me wondering “so what does it actually mean?”

The conclusion section at the end of the discussion is empty!

Minor comments:

- Throughout the manuscript, some paragraphs are extremely short, sometimes even only one sentence. Consider to integrate into adjacent paragraphs.
- Author summary: consider rephrasing last sentence which has “future” in it twice.
- Introduction, fourth paragraph: consider reversing the order in which the treatments are explained (i.e. set them in chronological order) such that the “(still do today)” is not needed.
- Methods, data section:
  - please clarify whether the HAT Atlas case data includes information about how cases were detected (passive vs. active screening) and whether that information was used when aggregating case numbers to the health zone level.
- Figure 1 suggests that there is no transmission in Kinshasa, whereas Figure 2 suggests that after all cases were reported there. It would be good to be explicit about whether or not inference was done for Kinshasa.
- Table 2: it took me a while to under what was meant by “Relative treatment rate from stage 2 factor, pre-1998”, which I only got after reading the next line “Treatment rate from stage 2, pre-1998” and the associated definition in the “percentiles” column (which is not a very logical place for this information). Suggestion: switch the two rows and put the definition of “Treatment rate from stage 2, pre-1998” in the “prior” column. Also, consider removing the symbol “X” and tilde sign which are used in all rows of the column “prior”; I got confused for a moment by the use the same symbol for all parameters.
- Lines 144-147: parameters s_1 and s_4 are not mentioned in either Table 1 or 2.
- Lines 195-196: rephrase “…which is especially important as there become very few cases remaining.”
- Log-likelihood function: index *i* is used here to indicate time (year) whereas in the equations it’s used to indicate risk group; I suggest to use index *t* for time to avoid confusion.
- Lines 222-225: I don’t understand the need for a negative binomial likelihood; why not use the regular likelihood as for the rest of the data points and simply drop the terms associated with screening if that did not happen?
- Line 256: I hope that during the sampling phase, the proposals were no longer drawn adaptively! The text suggests that adaptive proposals were still being done.
